# A caste differentiation mutant elucidates the evolution of ant social parasites

**DOI:** 10.1016/j.cub.2023.01.067

**Published:** 2023-02-28

**Authors:** Waring Trible, Vikram Chandra, Kip D. Lacy, Gina Limón, Sean K. McKenzie, Leonora Olivos-Cisneros, Samuel V. Arsenault, Daniel J. C. Kronauer

**Affiliations:** 1 Laboratory of Social Evolution and Behavior, The Rockefeller University, 1230 York Avenue, New York, NY 10065, USA; 2 John Harvard Distinguished Science Fellowship Program, Harvard University, 52 Oxford Street, NW Cambridge, MA 02138; 3 Department of Organismic and Evolutionary Biology, Harvard University, 52 Oxford Street, Cambridge, MA, 02138, USA; 4 Department of Microbiology, New York University School of Medicine, 430 E. 29^th^ Street, New York, NY, 10016, USA; 5 Oxford Nanopore Technologies, Oxford, OX4 4DQ, UK; 6 Howard Hughes Medical Institute, New York, NY 10065, USA; 7 Lead Contact

## Abstract

Most ant species have two distinct female castes, queens and workers, yet the developmental and genetic mechanisms that produce these alternative phenotypes remain poorly understood. Working with a clonal ant, we discovered a variant strain that expresses queen-like traits in individuals that would normally become workers. The variants show changes in morphology, behavior, and fitness that cause them to rely on workers in wild-type colonies for survival. Overall, they resemble the queens of many obligately parasitic ants that have evolutionarily lost the worker caste and live inside colonies of closely related hosts. The prevailing theory for the evolution of these workerless social parasites is that they evolve from reproductively isolated populations of facultative intermediates that acquire parasitic phenotypes in a stepwise fashion. However, empirical evidence for such facultative ancestors remains weak, and it is unclear how reproductive isolation could gradually arise in sympatry. In contrast, we isolated these variants just a few generations after they arose within their wild-type parent colony, implying that the complex phenotype reported here was induced in a single genetic step. This suggests that a single genetic module can decouple the coordinated mechanisms of caste development, allowing an obligately parasitic variant to arise directly from a free-living ancestor. Consistent with this hypothesis, the variants have lost one of the two alleles of a putative supergene that is heterozygous in wild types. These findings provide a plausible explanation for the evolution of ant social parasites and implicate new candidate molecular mechanisms for ant caste differentiation.

## eTOC

Trible *et al*. report a variant strain of the clonal raider ant that expresses queen-like traits in individuals that would normally become workers. These obligately parasitic variants have a mutated supergene and arose directly from their free-living parent strain, suggesting a genetic mechanism for the evolution of ant workerless social parasites.

## Introduction

Most ant species have two distinct morphological castes: queens and workers (Data S1). The developmental and genetic mechanisms that underpin these alternative phenotypes remain poorly understood^1,2^, however, and the study of caste development and evolution is largely limited to candidate genes from traditional model organisms, in part because it is impractical to isolate and characterize naturally occurring caste mutants using sexually reproducing ants.

**Figure 1.**
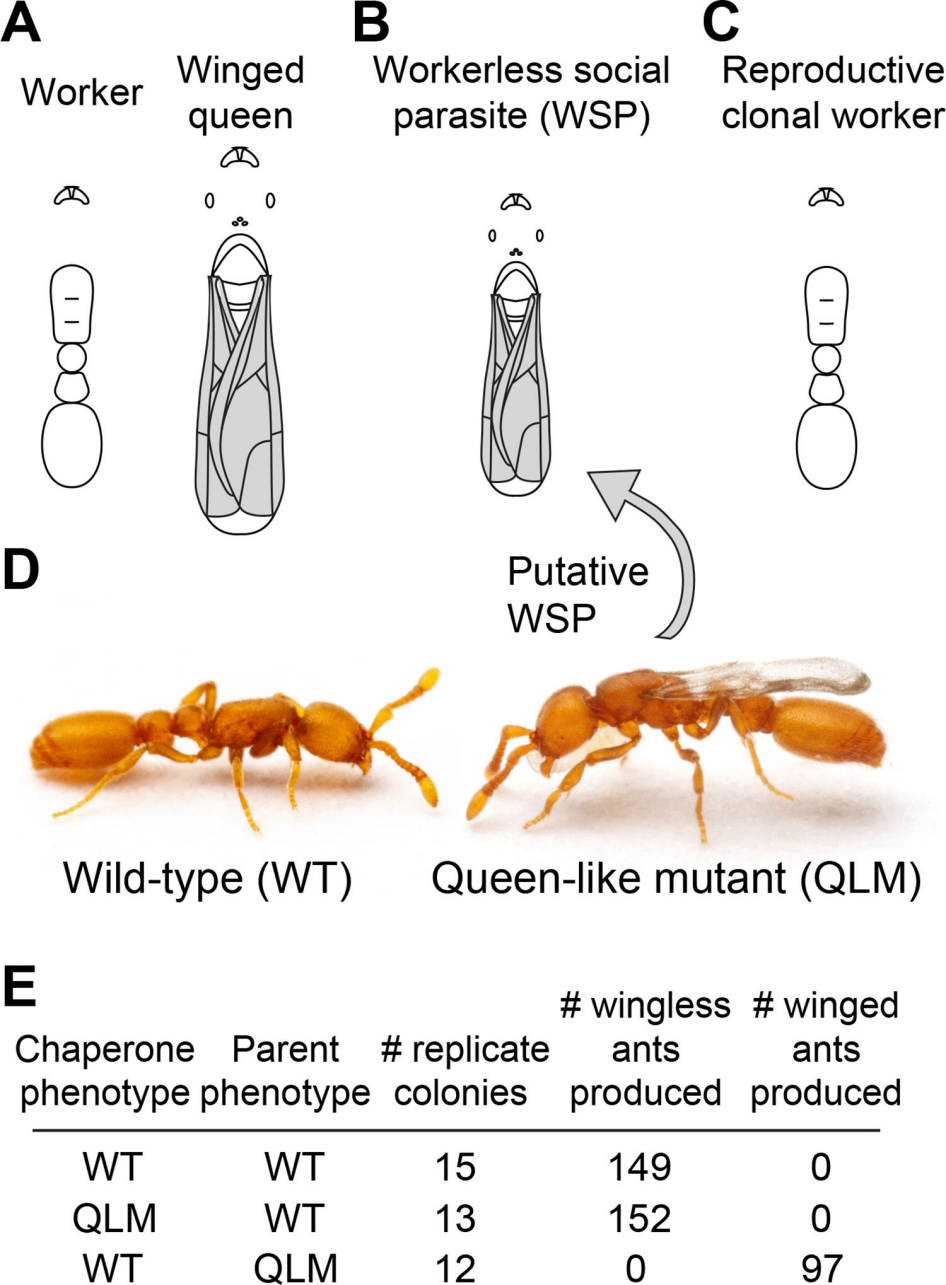
A queen-like mutant in the clonal raider ant. (A) Ancestral caste morphology in ants, exemplified by the raider ant *Ooceraea octoantenna*. Relative to workers, queens are larger and possess more developed reproductive, visual, and flight systems. (B) Workerless social parasite that expresses queen-like morphological features at a worker-like body size, using a miniaturized *O. octoantenna* queen as a hypothetical example. (C) Caste morphology of WT *O. biroi*. Relative to other *Ooceraea* and the ancestral condition of ants, *O. biroi* has evolutionarily lost the morphological queen caste and instead has colonies that are exclusively composed of parthenogenetic worker ants. (D) Immature WT *O. biroi* (left) and QLM (right). (E) Cross-fostering shows that the QLM phenotype is 100% penetrant. Eggs were collected and hatched on glass slides, and colonies were established with 20 adult WTs and 16 young larvae. Cartoons in A-C traced from published images^36^. See also Table S1 and Data S1.

One of the most extreme forms of caste evolution occurs in the workerless social parasites that feature a modified and miniaturized queen morphology and loss of the worker caste (Figure 1, A and B)^1,3–5^. All workerless social parasites are obligate inquilines: they live inside host colonies that they exploit for food, shelter, and brood care, and they possess a suite of derived phenotypes, known as the inquiline syndrome, that facilitate this lifestyle^5–9^. Workerless social parasites exploit the colonies of closely related ants, which they resemble chemically and morphologically. In fact, the initial host of many workerless social parasites may be the free-living forms of their parental populations^4,10–12^. If we assume that complex parasitic phenotypes evolve gradually via genome-wide selection, the close phylogenetic relationship between parasites and their hosts implies that a newly emerging parasite must quickly develop reproductive isolation to evolve into a highly specialized, obligate workerless social parasite^10,13–16^.

In 1971, E.O. Wilson proposed a mechanism for the evolution of workerless social parasites based on the assumption that parasites cannot sympatrically speciate from their host^10^. He argued that facultative parasitism always evolves before obligate parasitism and the loss of the worker caste. Under this scenario, nascent parasites must be able to survive independently from their host/ancestor so that they can speciate in allopatry and then re-encounter the host and evolve into an obligate parasite. Wilson incorporated Ernst Mayr’s views of speciation into his framework, but earlier researchers, including William Morton Wheeler and Charles Darwin, also proposed that obligate ant social parasites evolve from facultative intermediates^4,10,11^. This facultative hypothesis predicts that any obligate parasites should be phylogenetically nested within a clade of facultative parasites, rather than being sister to a free-living ant. However, recent molecular phylogenetic evidence implies that most workerless social parasites arise directly from free-living ants, rather than evolving from facultative parasites as Wilson’s theory proposes^4^.

In recent years, researchers have discovered that many complex intraspecific polymorphisms are conferred by discrete genetic modules, including sex chromosomes, social chromosomes, and other supergenes^17–25^. These discoveries raise the alternative possibility that the suite of traits necessary for workerless social parasitism can be conferred by the inheritance or transformation of a single genetic locus, or module^26^. Under this scenario, a workerless social parasite could emerge as an intraspecific genetic variant within a free-living population due to the action of a single locus, while gene flow across the rest of the genome gets disrupted only secondarily. Therefore, the discovery of a pleiotropic genetic module that confers a viable parasitic phenotype could provide a solution to the mystery of the rapid, parallel, and sympatric evolution of ant workerless social parasites.

## Results

The clonal raider ant, *Ooceraea biroi*, is a queenless, asexual ant species in which wild-type (WT) individuals have attributes typical of wingless worker ants (Figure 1C)^27^. During extensive screening of laboratory colonies, we discovered a series of individuals that possessed prominent wings as pupae and immature adults (Figure 1D). Mature adults shed the wings but retain queen-like wing scars and associated segmentation of the mesosoma (Figure S1). These winged individuals caught our attention because no morphological queens or winged female adults have been reported for this species^28,29^.

To test whether this phenotype arises via genetic variation or phenotypic plasticity, we isolated winged individuals and wild-type controls for rearing experiments that featured cross-fostering of the two types. Eggs from winged individuals reared by WTs developed into winged individuals with 100% penetrance. In contrast, WT eggs reared by winged individuals invariably developed into WT adults (Figure 1E). Furthermore, a series of randomly selected winged individuals had multilocus microsatellite genotypes identical to those of Clonal Line A, the WT clonal genotype of the source colony C1 in which the variants were discovered (Table S1)^28^. These results indicate that the winged individuals are not products of phenotypic plasticity but represent a genetic variant of Clonal Line A that, as with workerless social parasites, constitutively develops wings. We therefore designated these ants as a queen-like mutant (QLM) variant.

In principle, there are two developmental genetic mechanisms by which a queen-like morphology could re-appear in a queenless ant species (see Data S1 and Figure S2 for details). In most ant species, morphological caste is an epigenetic outcome of phenotypic plasticity and is tightly coordinated with overall body mass: larger individuals develop wings and a suite of additional queen-like morphological traits, while smaller individuals develop worker-like morphology^1,5^. Some ant lineages display reduced phenotypic plasticity and exhibit a genetic bias for queen development^30,31^. For instance, a ‘cheater’ genotype with queen morphology has been reported from a different queenless clonal ant species (Figure S2)^32^. In this and all other previous reports of genetic biases for queen development, the resulting adults are large-bodied and phenotypically indistinguishable from queens induced via phenotypic plasticity, and rare individuals that metamorphose at low body mass develop into adults with typical worker morphology (Data S1, Figure S2). This indicates that the underlying genotypes do not directly induce queen-like differentiation of tissues, but instead indirectly induce queen development via an influence on body size (Data S1, Figure S2)^1,3,33^. In other words, they affect caste determination (i.e., the probability that a larva will develop into a queen), but do not alter the association between overall body size and adult caste morphology^1,30,31^. This mechanism thus results in phenotypically normal queens that are larger than workers.

The second potential mechanism is inspired by the many workerless social parasites that display queen-like phenotypes at worker-like body size (Figure 1B)^5,7–9^. These phenotypes are distinct from those of typical queens and cannot result directly from changes to the body size distribution (Figure S2). Instead, these workerless social parasites display altered caste differentiation, the allometric scaling mechanisms that coordinate tissue growth with overall body mass^1,3,7^. Therefore, queens produced by genetic changes to caste differentiation should display an abnormal association between body size and adult caste morphology (Figure 1B)^1,3,6,8,34^.

**Figure 2.**
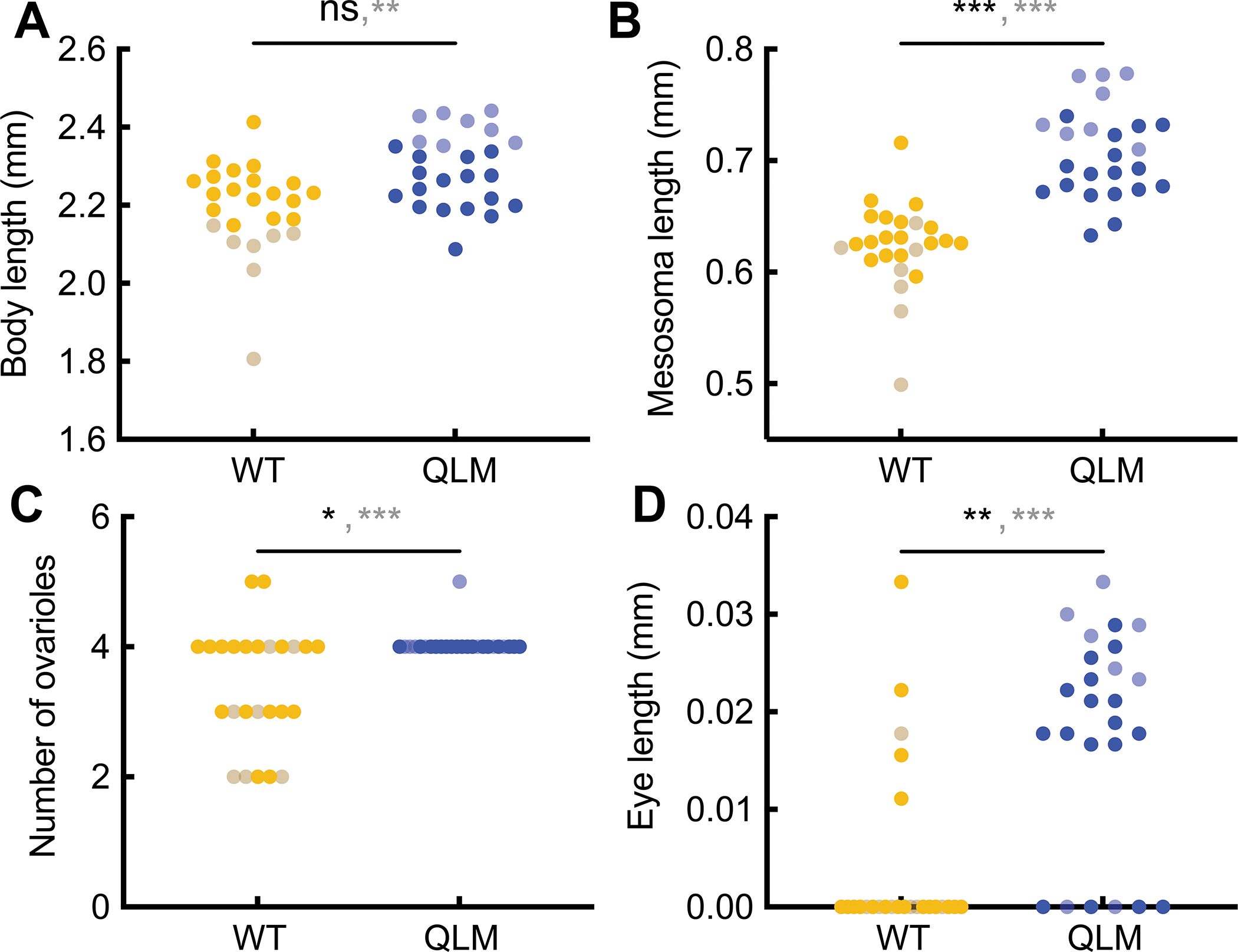
QLMs and WTs overlap in size but differ in caste morphology. (A) Body length of WT and QLM adults. The size distribution of WTs and QLMs is largely overlapping, but QLMs are larger on average. (B) Mesosoma length of WT and QLM adults. QLMs have a longer mesosoma, reflective of their wing development. (C) Ovariole number of WT and QLM adults. QLMs have significantly more ovarioles than WTs. (D) Eye length of WT and QLM adults. QLMs have significantly larger eyes than WTs, most of which lack eyes entirely (Figures S1 and S3). Two p values are reported for each panel: opaque text corresponds to the dataset with individuals excluded to produce a size-matched population (opaque points; n = 18 WT and 17 QLM adults) and translucent text corresponds to the data with all individuals (translucent plus opaque points; n = 25 WT and 25 QLM adults). ns: not significant; *p<0.05; **p<0.01; ***p<0.001. p values from unpaired Wilcoxon tests. See also Figures S1–S3 and Data S2.

To distinguish these alternatives in *O. biroi* QLMs, we collected morphometric data from WTs and QLMs reared under identical conditions (Data S2A). Although the two types show overlap in the distribution of overall body size, QLMs are significantly longer than WTs on average (Figure 2A). QLMs are also queen-like in other features: they have a longer, more segmented mesosoma (Figure 2B, Figure S3, A and B), more ovarioles (Figure 2C, Figure S3C), and more developed eyes (Figure 2D, Figure S3D) than WTs.

To test whether QLMs display an altered relationship between body size and caste morphology indicative of a change to caste differentiation, we sub-sampled our initial morphometrics dataset to produce a size-matched population of WT and QLM adults. Although QLMs in this subset do not differ from WTs in body length, they retain significantly greater mesosomal length, ovariole number, and eye length than WTs (opaque dots in Figure 2 and Figure S3, A–D). These QLMs also have relatively smaller heads and petioles than WTs (Figure S3, E and F). All of these morphological features have been observed in workerless social parasites (Figure S2)^5,30,31^.

**Figure 3.**
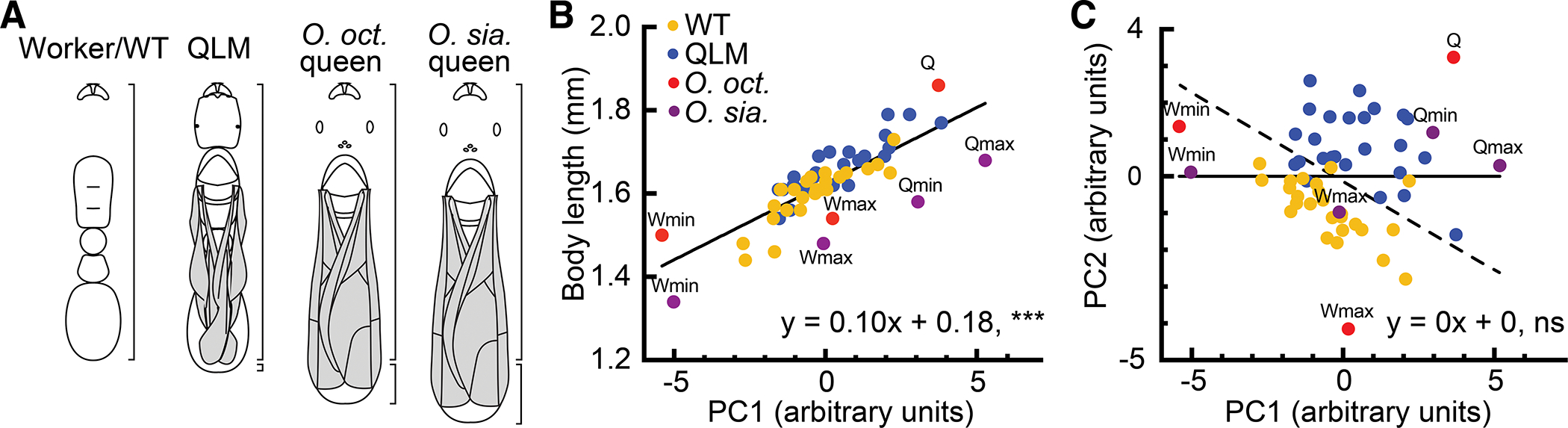
The size overlap between WTs and QLMs is not characteristic of typical *Ooceraea* queens. (A) Cartoons of a QLM, an *O. octoantenna* queen, and an *O. siamensis* queen rescaled to the average worker size of their respective species. QLMs are 4.2% longer than WTs, on average, while *O. octoantenna* and *O. siamensis* queens are 15.6% and 22.4% longer than workers, respectively (lower brackets to the right of each cartoon show the length increase). (B) Linear regression of PC1 and body length for all samples. Workers of *O. octoantenna* (worker minimum, worker maximum, and one queen) and *O. siamensis* (worker minimum, worker maximum, queen minimum, and queen maximum) have lower body length and PC1 values than queens of their respective species, and workers and queens do not overlap in size. PC1 and body length are correlated, indicating that PC1 is positively associated with body size. (C) Linear regression of PC1 and PC2 for all samples. A diagonal line (dashed) can separate samples into workers and WTs (lower left) versus queens and QLMs (upper right). ns: not significant; ***p<0.001. p values from linear regression with an expected slope of zero. Equations provide best-fit slope and y-intercept. *O. sia.*: *Ooceraea siamensis*, *O. oct.*: *Ooceraea octoantenna*. See also Figures S1–S3 and Data S2.

To assess morphological similarities between QLMs and the ancestral queen caste of *O. biroi*, we performed a principal components analysis using workers and queens of two close relatives of *O. biroi* that have retained the queen caste, *Ooceraea octoantenna* and *Ooceraea siamensis* (Data S2B and C)^35,36^. Both *O. octoantenna* and *O. siamensis* queens are larger than the largest workers of their species (Figure 3A and B), with queens averaging 22.4% and 15.6% longer than workers, respectively. While *O. biroi* QLMs are only 4.2% longer than WTs, on average (Figure 3A and B), a plot of the first two principal components of the morphometric dataset revealed that *O. biroi* QLMs group with heterospecific queens, while *O. biroi* WTs group with heterospecific workers (Figure 3C). These results confirm that *O. biroi* WTs are homologous to the workers of other *Ooceraea* species, and that *Ooceraea* queens are distinctly larger than workers. In contrast, QLMs display a novel morphological syndrome involving queen-like phenotypes at worker-like body size. This indicates that the QLMs do not constitute an atavism that restores the ancestral queen phenotype of *Ooceraea*, but instead represent a novel phenotype that resembles the miniature queens of workerless social parasites more closely than any other form of ant natural history variation (Figure S2). Our cross-fostering and morphometric results thus demonstrate that genetic variation in caste differentiation can simultaneously induce worker-sized queens and prevent production of the worker caste.

**Figure 4.**
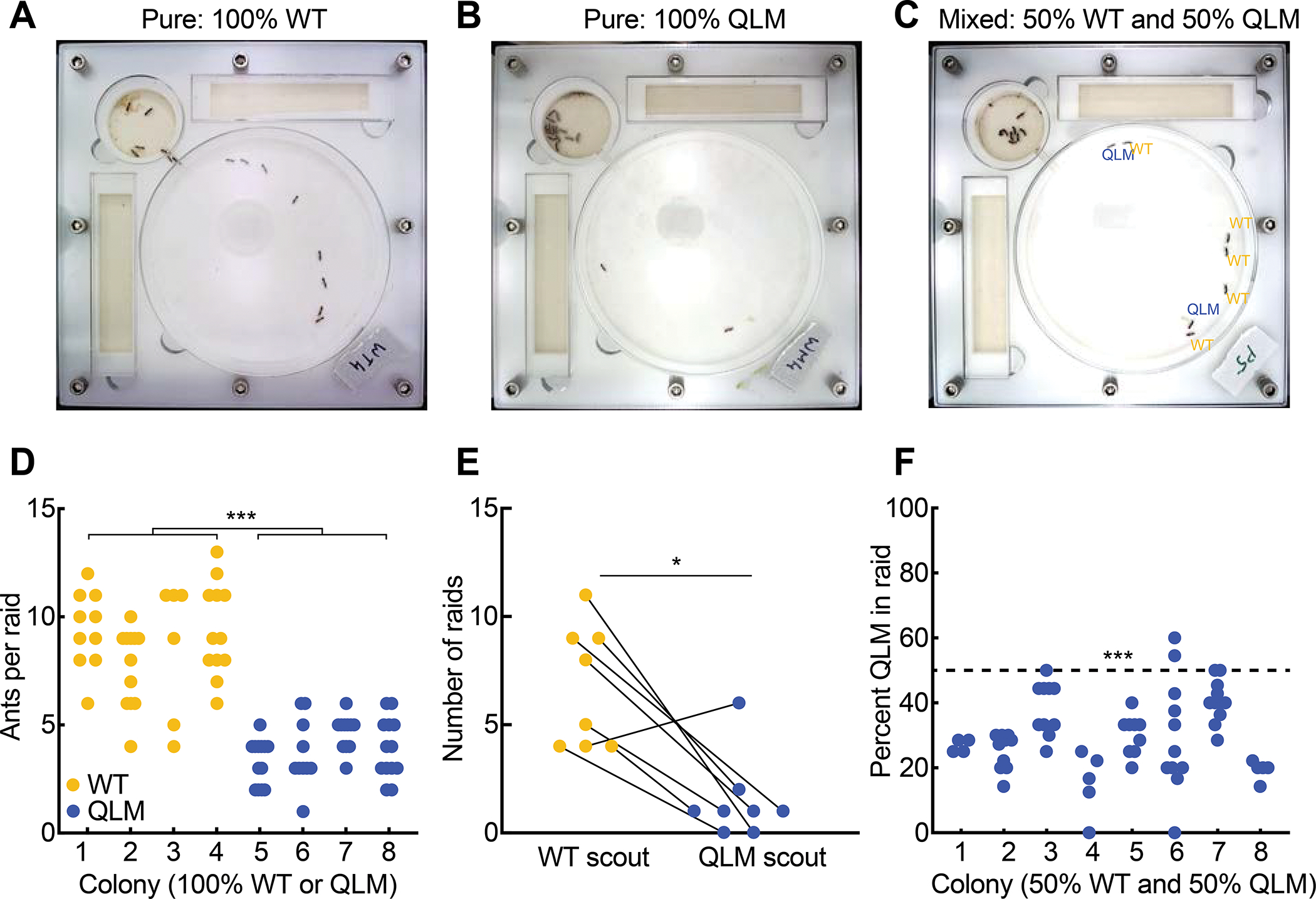
QLMs show a reduced tendency to forage. (A-C), Images taken at peak of raiding activity in representative pure WT (A), pure QLM (B), and mixed (C) colonies (n = 16 ants/colony). (D) Number of ants per raid in pure WT and QLM colonies (n = 4 colonies composed of 100% WT or 100% QLM adults; n = 40 WT and 47 QLM raids). (E) Number of raids initiated by WT and QLM scouts in each mixed colony. Lines connect number of WT- and QLM-initiated raids for each of n = 8 colonies composed of 50% WT and 50% QLM adults. A total of 54 and 12 raids were initiated by WT and QLM scouts, respectively. (F) Fraction of QLMs among raid participants in mixed colonies. Dashed line indicates the null-expectation if the two morphs are equally likely to participate in raids. *p<0.05; ***p<0.001. p values from unpaired (D) or paired (E) two-way Wilcoxon tests, or from a one-way Wilcoxon test (F) against an expectation of 50% QLMs in raids. See also Figure S4 and Videos S1–S2.

The morphological worker caste is typically also the specialized behavioral foraging caste in ants, while the morphological queens of free-living and inquiline species are specialized for reproduction and, in most species, do not forage^37,38^. Worker foraging in *O. biroi* takes the form of a multi-step raiding program^39^. Once an *O. biroi* scout worker has encountered prey, it lays a pheromone trail back to the nest, where it recruits a raiding party using a recruitment pheromone. The raiding party then follows the pheromone trail to the prey (Video S1)^39^. To test whether QLMs differ from WTs in this behavior, we set up experimental colonies of 16 age-matched ants, composed either of all WTs, all QLMs, or a 50:50 mix of the two variants, and measured multiple aspects of *O. biroi* raiding behavior (Figure 4, A–C). Pure colonies of QLMs conducted a similar number of raids as pure WT colonies, but QLM raiding parties were significantly smaller (mean of 8.75 WT versus 3.87 QLM ants per raid) (Figure 4D, Figure S4A, Video S1, Video S2). In mixed colonies, QLM scouts initiated significantly fewer raids than WT scouts (Figure 4E; WT scouts: 6.75 raids on average; QLM scouts: 1.5 raids on average), and QLMs were under-represented among the ants that participated in the raids (Figure 4F; 29.3%, on average). The genotype of the scout was not associated with the number of ants participating in the raid, implying that the rare QLM scouts show normal recruitment behavior (Figure S4B). These results demonstrate that the underlying genetic factors that produce queen-like morphology also produce a reduced tendency to engage in worker-like foraging behavior.

Lineages that replace workers with worker-sized queens have evolved over a dozen times in ants, suggesting that a wide phylogenetic range of ant species have the capacity to evolve into workerless social parasites^4^. Importantly, every described ant species that has lost the morphological worker caste is an obligate inquiline parasite, and typically occurs at a low frequency in the populations of its host^4^. To test whether the QLMs can function as parasites of WT colonies, we collected fitness data at multiple stages of the *O. biroi* life cycle.

**Figure 5.**
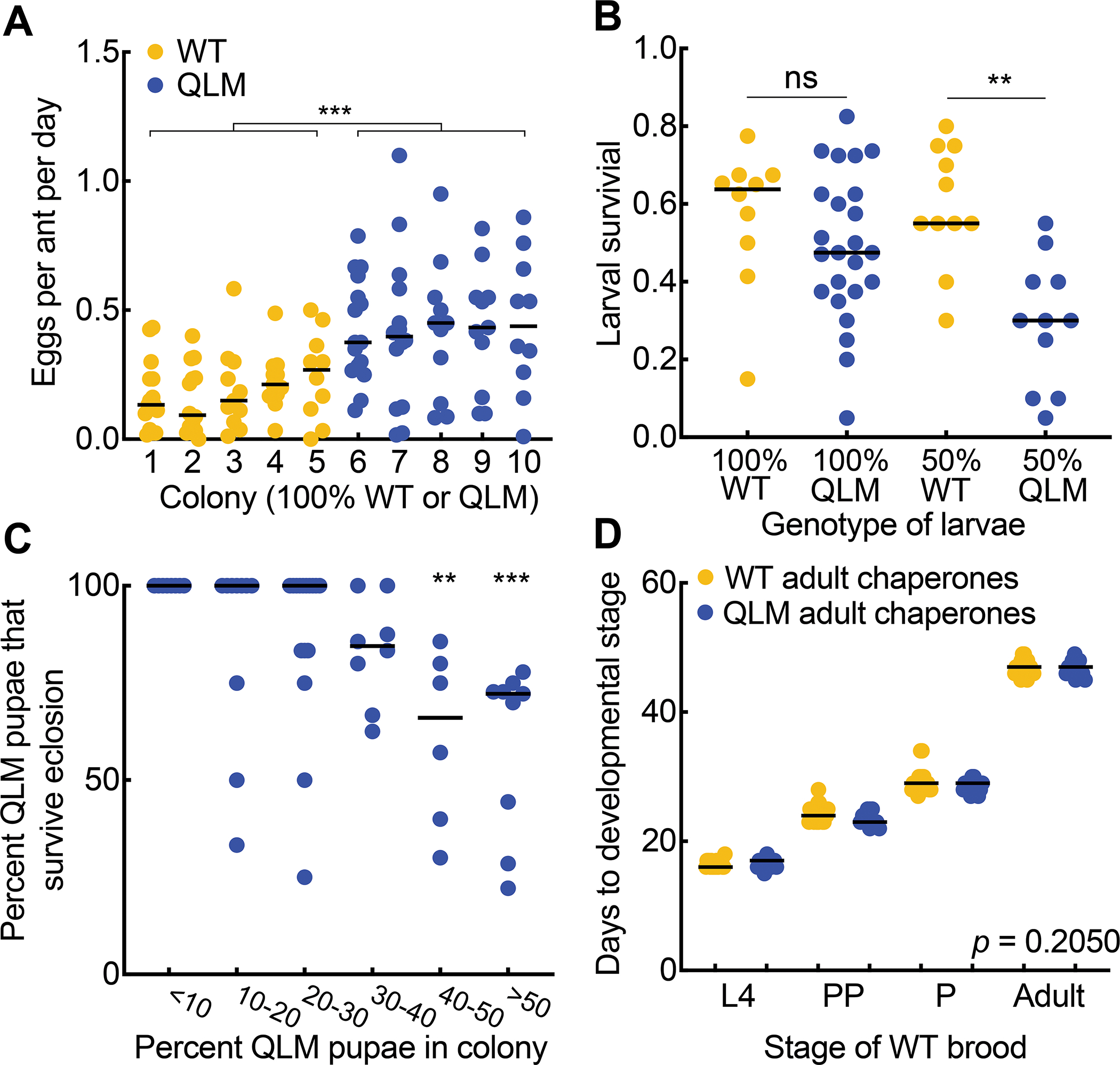
QLMs have both positive and negative shifts in fitness. (A) Egg-laying rate in WT and QLM colonies (n = 5 colonies and 62 paired collection days per genotype). (B) Egg to pupa survival of brood reared by WT adults (n = 26–40 eggs per colony). Brood were 100% WT (n = 10 colonies), 100% QLM (n = 24 colonies), or 50% WT and 50% QLM (n = 11 colonies; WT and QLM survival are reported separately for each colony). (C) Percent of QLMs that survive eclosion (pupa to adult transition). QLMs were reared by WT adults in colonies with varying percentages of QLM vs. WT pupae (n = 57 colonies and 6–33 pupae per colony). (D) Developmental periods for different stages in WT individuals (n = 16 larvae per colony) reared by colonies of WT or QLM adults (n = 19 WT and 13 QLM colonies). ns: not significant; **p<0.01; ***p<0.001. p values from unpaired ((A), comparison of pure WT and QLM colonies in (B)) or paired (comparison of WT and QLM survival in mixed colonies in (B)) two-way Wilcoxon tests, or from one-way ((C); percent of pupae) or two-way ((D); developmental stage and colony type) ANOVA followed by Tukey’s test. Black bars depict sample means. In all fitness experiments, colonies consisted of 20 WT or QLM adults. See also Figure S5.

Consistent with their elevated ovariole number, QLMs laid approximately twice as many eggs as WTs, on average (Figure 5A; 0.43 eggs/QLM/day versus 0.19 eggs/WT/day). Cross-fostering these eggs into matched rearing units revealed that the QLMs exhibit a competitive disadvantage as larvae: their survival to pupation did not differ significantly from WTs when all eggs in the colony were QLMs but was significantly lower than WT survival in colonies established with 50% WT and 50% QLM eggs (Figure 5B).

While rearing the QLMs, we did not observe any aggression from the WTs, but noticed that they frequently died during eclosion. This might be due to an increased difficulty in molting imposed by their bulky wings, as the dead QLMs often had their pupal skin only partially removed. To quantify this effect, we established colonies in which WT adults reared cohorts of pupae that varied in the percentage of QLMs. In colonies with 10% or fewer QLM pupae, survival of QLM pupae was 100% (Figure 5C, Figure S5A and B). In contrast, the survival of QLMs decreased as their frequency increased, with approximately 50% survival when more than half of the pupae were QLMs (Figure 5C, Figure S5, A and B). The survival of WT pupae was 98.5% and did not vary as a function of the percentage of WT pupae in a colony (Figure S5C). Finally, we studied the ability of QLMs to rear larvae and did not observe any difference in the development time (Figure 5D) or survival (Figure S5D; mean = 80.8% versus 72.7% survival, respectively; p = 0.3184) of WT larvae reared by WT or QLM adults.

These results indicate that QLMs have phenotypes with both positive and negative fitness consequences. In colonies with a minority of QLMs, these fitness effects are balanced such that the proportion of QLMs produced is not significantly different from their frequency in the colony (Figure S5E). In conjunction with our morphometric and behavioral studies, these results show that the QLMs, like workerless social parasites, can function as inquilines of WT colonies^5,40^. The QLMs therefore provide a unique opportunity to investigate the genetic architecture of social parasitism.

**Figure 6.**
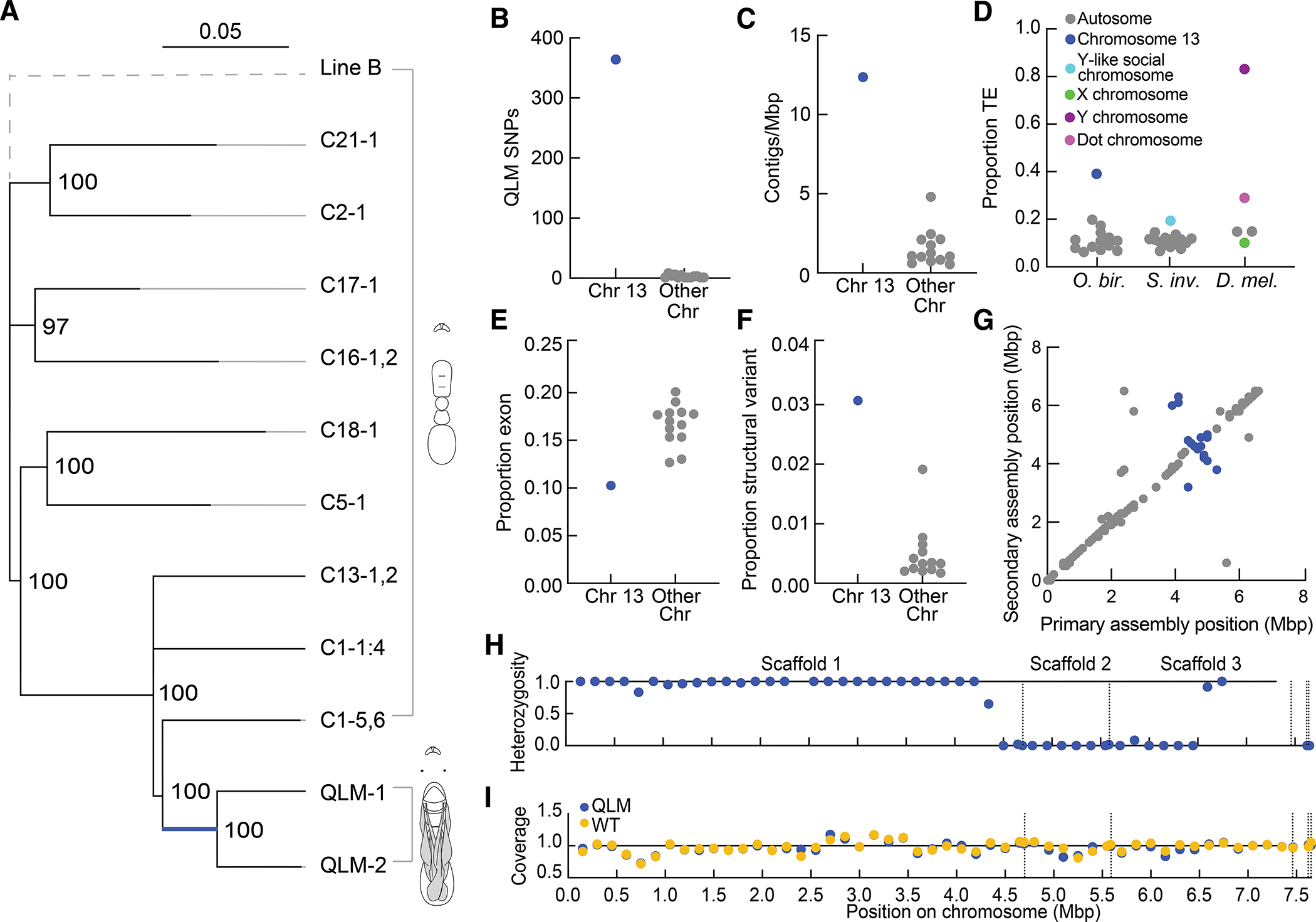
QLMs have a large loss of heterozygosity in a putative supergene. (A) Maximum likelihood phylogeny from whole genomes of 15 WT Clonal Line A ants and two QLMs, with a single Clonal Line B ant as an outgroup (grey). Clades of WT ants from the same colony are collapsed. Numbers at nodes represent bootstrap support from 100 replicates, and the scale bar indicates 5% divergence among polymorphic sites across the dataset. Nodes with less than 90% bootstrap support are collapsed. (B) Number of SNPs distinguishing QLMs from WTs on Chromosome 13 versus all other 13 chromosomes. These SNPs correspond to the heavy blue bar on the phylogeny in (A). (C) Contigs per megabase (Mbp) for *O. biroi* chromosomes. (D) Proportion TE for chromosomes in three insect species. Chromosome 13 of *O. biroi* has high TE content, similar to the Y-like social chromosome of fire ants, and the non-recombining Y chromosome and dot chromosome of fruit flies, but unlike the X chromosome or autosomes. (E) Proportion exon for *O. biroi* chromosomes. (F) Proportion structural variant for *O. biroi* chromosomes. (G) Sequence alignment dot plot of the primary and secondary assemblies of Chromosome 13. The contigs of the secondary assembly were scaffolded by aligning them to the primary assembly, so this plot is expected to provide a minimum estimate of any differences in the spatial organization of the Chromosome 13 haplotypes. In spite of this, the region from 3.9–5.3Mbp in the primary assembly (blue points) is partially inverted and partially translocated in the secondary assembly. (H) Relative heterozygosity per 250kbp window of Chromosome 13 in QLMs in our improved Chromosome 13 assembly. Heterozygosity is shown as the fraction of heterozygous sites in the WT sister clade (C1–5 and C1–6) that are also heterozygous in QLMs. The few sites at which QLMs apparently retain heterozygosity are likely spurious and arise in instances where reads from duplicate genomic loci were mis-mapped to a single locus in the reference assembly. Windows without data are homozygous in the WTs. Our final Chromosome 13 assembly, v5.7, consists of three large scaffolds and three small unplaced contigs (on the right; dotted vertical lines). (I) Normalized sequencing read depth per 250kbp window averaged across both QLMs and the two genomes in the WT sister clade (C1–5 and C1–6). (A-F) and (G-I) are based on the Obir v5.4 and v5.7 genome assembly, respectively. *O. bir.*: *Ooceraea biroi*, *S. inv*.: *Solenopsis invicta*, *D. mel*.: *Drosophila melanogaster*. See also Figure S6 and Data S3.

To better understand the genetic event that induced the QLM phenotype, we conducted whole-genome short-read resequencing of individual ants at 35X coverage (range: 27.4 to 41.5) for two QLMs and 15 WT Line A adults and used this dataset to create a whole-genome molecular phylogeny (Figure 6A, Data S3A). QLMs are monophyletic and nested within the genetic diversity of colony C1, the colony in which they were discovered (Figure 6A). Given that the species reproduces clonally and we did not observe any phenotypic intermediates between WTs and QLMs in C1 (or any other colony), this phylogenetic result indicates that the QLMs arose from a WT ancestor due to a recent mutation in a clonal sub-lineage of colony C1.

Mapping the causal genetic basis for this phenotype is challenging because *O. biroi* is parthenogenetic, and we are therefore not able to perform crosses for a traditional association study. However, we can assume that all QLMs have the same genotype at any loci that are causal to the phenotype, and that this causal genotype is not found in any WT Clonal Line A genomes. Of 168,819 single-nucleotide polymorphisms (SNPs) in our short-read dataset, only 400 SNPs differed consistently between QLMs and WTs. Interestingly, 364 of these SNPs (91%) mapped to the second-smallest chromosome in *O. biroi*, Chromosome 13 (Figure 6B). Of the SNPs on Chromosome 13, all are homozygous in QLMs but heterozygous in WTs. This pattern likely arose via a single copy-neutral loss of heterozygosity across a segment of the chromosome, which can arise in parthenogenetic organisms that reproduce via post-meiotic fusions due to the inclusion of a recombined and a non-recombined copy of a homologous chromosome in the zygote^41^. Such a loss of heterozygosity can also arise via mitotic recombination, as observed in a substantial portion of human cancers^42^.

The 36 SNPs outside of Chromosome 13 that distinguish QLMs from WTs can be ascribed to 28 separate mutations: 9 *de novo* SNPs and 19 small losses of heterozygosity (Data S3B). Based on prior estimates from related species, we would expect to see approximately 1.6 *de novo* SNPs across the genome each generation (assuming a mutation rate of ca. 3.5 × 10^−9^ / haploid genome / generation^43,44^ and a haploid genome size of 224 Mbp in *O. biroi*^45^). The 9 *de novo* SNPs that distinguish QLMs from colony C1 WTs therefore imply that the QLMs originated in our lab culture only a few generations ago. In light of this recent origin and the very small number of mutations that distinguish the QLM lineage from WTs (including SNPs, indels, and losses of heterozygosity; Data S3B), it is probable that a single mutational event induced the QLM phenotype, rather than that it arose via a gradual series of mutations.

We manually inspected all loci with genotypes unique to QLMs outside of Chromosome 13 and did not detect any compelling candidates (Data S3B). Chromosome 13, on the other hand, is unusual in that it is heteromorphic in the *O. biroi* karyotype and contains by far the highest density of contigs in the *O. biroi* genome assembly (Figure 6C)^45,46^. Chromosomes with similar features are known to underlie alternative phenotypes in many animals, including sex chromosomes and the supergenes that regulate social organization in multiple species of ants^24,25,47^. Using further bioinformatic characterization, we found that Chromosome 13 is enriched for transposable elements and low in proportionate exon content, similar to the fire ant social chromosome and the Y chromosome of *Drosophila melanogaster* (Figure 6, D and E). These sequence characteristics are found in WT Line A ants, implying that the supergene-like features of Chromosome 13 were present before the mutational origin of the QLMs. However, we currently cannot determine precisely when or how these features evolved (see Discussion).

To determine the location and contents of the loss of heterozygosity, we used a haplotype-aware algorithm to re-assemble published sequencing data with higher contiguity into a diploid assembly, Obir v5.6. We then used alignments of multiple *de novo* assemblies to further improve the assembly of Chromosome 13, yielding a final Obir v5.7 assembly (see STAR Methods for details). This resolved Chromosome 13 into three large, ordered scaffolds and three unplaced small contigs, compared to 116 contigs unplaced within the chromosome in the previously published assembly, Obir v5.4 (Data S3C)^45^. Comparing the primary and secondary contigs of this haplotype-aware assembly revealed that Chromosome 13 is enriched for structural variants (defined as indels 50bp or greater in length) and harbors at least one large inversion (Figure 6, F and G). These results indicate that Chromosome 13 contains a putative supergene, similar to the sex chromosomes and social chromosomes that regulate complex phenotypes in many animals^20,24,25,47^.

The loss of heterozygosity in QLMs can be explained by a recombination event spanning 2.25 Mbp of Chromosome 13 (Figure 6H, Data S3C). QLMs do not differ from WTs in sequencing depth within this region, indicating that the homozygosity on Chromosome 13 indeed represents a copy-neutral loss of heterozygosity rather than a segmental deletion (Figure 6I, Figure S6). At each site in the focal region, the QLM sequence is identical to one of the parent WT haplotypes, showing that it did not accumulate mutations that might contribute to the phenotype subsequent to the recombination event that produced the loss of heterozygosity. We identified the start and stop positions of the loss of heterozygosity but did not find evidence for a loss-of-function mutation caused by the recombination event (Data S3D). The loss of heterozygosity spans 81 structural variants (total length: 98,208 bp) that are heterozygous in WTs and have either become homozygous or lost in the QLMs (Data S3E). In light of this large degree of sequence divergence, and in conjunction with the fact that supergenes with similar features regulate complex phenotypes in other animals, we hypothesize that the loss of heterozygosity event on Chromosome 13 induced the QLM phenotype. However, given that 28 additional mutations elsewhere in the genome also distinguish QLMs from WTs (Data S3B), we cannot formally rule out the possibility that the QLM phenotype was produced by another variant.

**Figure 7.**
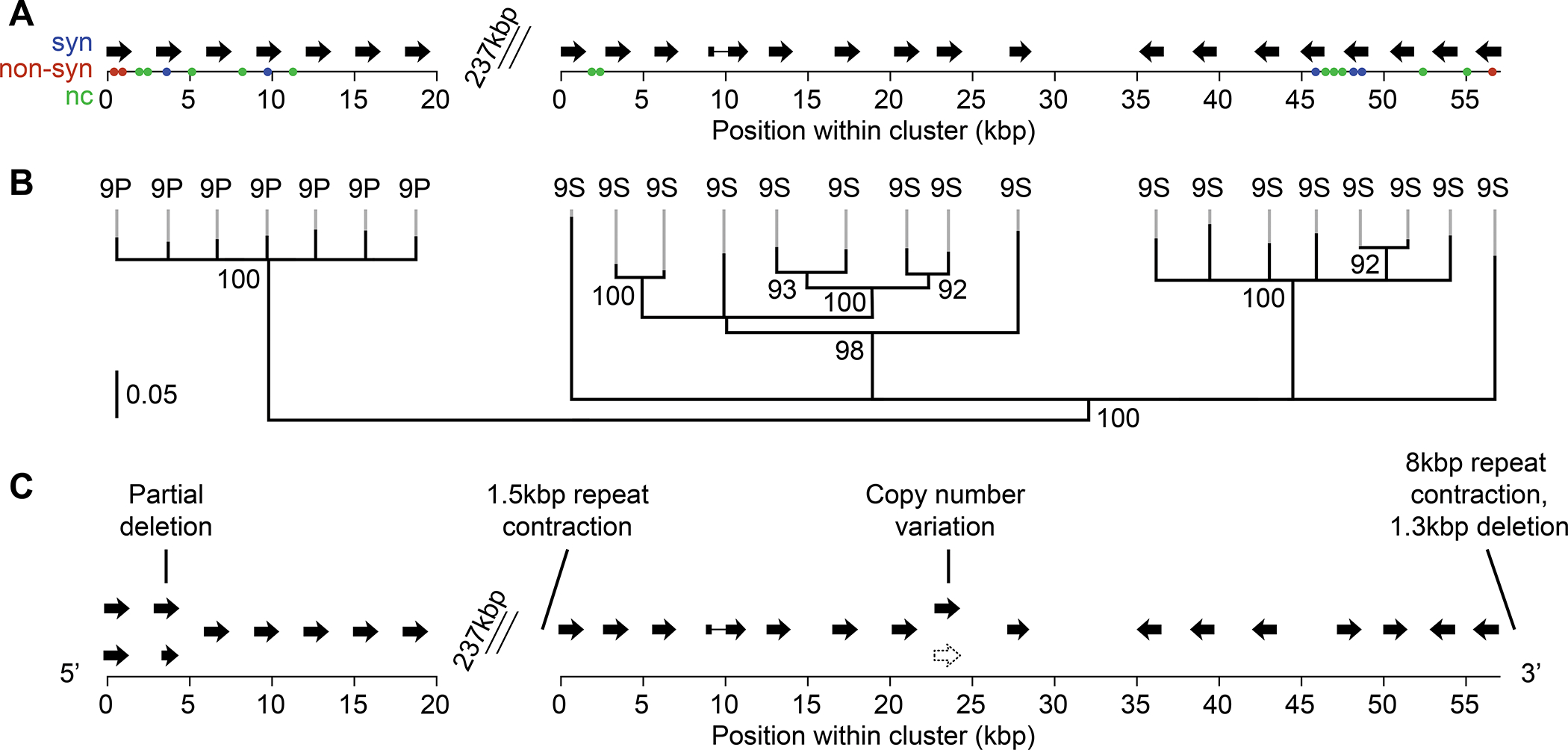
QLMs differ from WTs in two clusters of *CYP9* genes. (A) *CYP9* gene clusters in the Obir v5.4 assembly. Colored dots indicate heterozygosity in WTs in amino acid sequence (aa), synonymous substitutions (syn), and non-coding sequence (nc); all of these polymorphisms are homozygous in QLMs (Data S3B). In total, 22 sequence variants are associated with the 24 *CYP9* genes, a significant enrichment relative to the 48 variants affecting the remaining 176 genes in the loss of heterozygosity region (p < 0.001, Fisher’s exact test) (Data S3F). (B) The loss of heterozygosity region encodes seven CYP9P and 17 CYP9S proteins (see Figure S7 for identification of gene families within these CYPs). Node labels represent bootstrap support with 100 replicates, and scale bar represents 5% divergence at informative sites. Nodes with less than 90 bootstrap support were collapsed. (C) Structural variation between haplotypes of the *CYP9* gene clusters, inferred using a *de novo* annotation of the primary and secondary sequence of the Obir v5.6 diploid assembly (Data S3E). The repeat contraction at the 5’ end of the *CYP9S* cluster is 15kbp from the start codon of the first *CYP9S* gene, while the repeat contraction and deletion at the 3’ end of the array is 8kbp from the start codon of the final *CYP9S* gene. All of these haplotype differences are heterozygous in WTs but have become homozygous in QLMs. See also Figure S7 and Data S3.

This loss of heterozygosity region spans 186 genes in the *O. biroi* genome annotation (Data S3F). Within this gene set, the two most highly enriched GO terms are ‘oxidoreductase activity’ and ‘metabolic process’ (Data S3G), due to a number of *cytochrome P450 9* (*CYP9*) genes (Figure 7). The *CYP9* family is strongly overrepresented in the loss of heterozygosity region (p = 10^−20^, Fisher’s exact test using KEGG ortholog groups) (Data S3H). CYPs play essential roles in insect metamorphosis^48–52^. Many uncharacterized insect CYPs are expected to participate in hormone synthesis and metabolism, and ant CYP9s in particular are predicted to bind the molting hormone ecdysone^52–55^. Manual re-annotation showed that the loss of heterozygosity contains 24 of the total 27 *O. biroi CYP9* genes (Figure 7B, Figure S7). The loss of heterozygosity altered the genotype of QLMs relative to WTs in numerous features of the *CYP* array, including amino acid sequences, copy number, and adjacent non-coding sequences of *CYPs* (Figure 7, A and C, Figure S7, Data S3F). In ants, adult caste phenotypes arise during metamorphosis and are controlled via hormone signaling^1,2^. Thus, the CYP9 enzymes identified here constitute compelling candidate proteins for caste differentiation and other phenotypes of workerless social parasites.

## Discussion

Ant queens have fully formed reproductive, visual, and flight systems that are similar to those of their wasp ancestors, while ant workers develop according to a modified program that leads to reduced growth of these tissues during metamorphosis^1,2,34,56^. In other organisms, genetic polymorphisms that reduce or eliminate novel developmental programs (especially alternative phenotypes) are evolutionarily common^57,58^. Early population geneticists assumed that highly pleiotropic genetic variants are generally harmful, but more recent theory suggests that pleiotropic variants, including alleles introgressed via hybridization, can accelerate phenotypic evolution^22,23,59,60^. Our research adds to the evidence that even complex phenotypic syndromes can have a modular genetic and developmental organization that enables rapid evolution^18,19,61–63^.

The QLMs arose from WTs just a few generations before we discovered them in our laboratory colonies. We did not observe any intermediate phenotypes in the source colony, further supporting the conclusion that the QLM phenotype was not gradually assembled across multiple successive generations of clonal evolution. Instead, it seems that the phenotype was induced in a single genetic step: a WT parent produced a daughter with a phenotype resembling that of workerless social parasites. In a sexual ant species, an analogous event could immediately give rise to obligate workerless parasitism without the prior emergence of genome-wide genetic isolation. This raises the possibility that further divergence, including sympatric speciation and morphological, physiological, and behavioral aspects of the inquiline syndrome that are not present in QLMs^6,8,64^, may follow the evolution of obligate parasitism, rather than precede it. However, there are many different paths a free-living species can take to become an obligate parasite^3,4^. Our conclusions apply specifically to the evolution of workerless social parasites because QLMs and workerless social parasites share two characteristics that are not found in any other parasitic forms: the loss of the worker caste, and a major shift in allometric scaling that produces queens roughly the size of workers in the ancestral population. Other forms of social parasitism, such as brood raiding, nest usurpation, and xenobiosis, are not comparable to workerless social parasites or QLMs.

The QLMs possess a 2.25Mbp loss of heterozygosity that could have produced the complex QLM phenotype, either by containing a single causal allele or a group of causal alleles in linkage. This loss of heterozygosity is located on Chromosome 13, which is structurally similar to the non-recombining social chromosomes of other ant species^19,62^. This suggests the possibility that Chromosome 13 contains a supergene that confers social parasitism. Relative to the recent origin of the QLMs, this putative supergene must be ancient, and likely segregated in the ancestral sexual population in the form of a recessive Mendelian polymorphism for free-living and workerless social parasite variants. For example, this population could have possessed a recessive *workerless* allele, such that *W/W* and *W/w* are free-living wild-types, while *w/w* are QLMs. Line A WTs would then be heterozygous for this supergene (*W/w*), and the loss of heterozygosity produced a variant strain that is homozygous recessive (*w/w*) along some or all of the supergene.

*O. biroi* is parthenogenetic, so we cannot test for two key features of supergenes: Mendelian inheritance of the QLM phenotype and a suppression of recombination between alternative haplotypes. However, several pieces of evidence support a supergene origin for the QLM phenotype. First, Chromosome 13, unlike any other chromosome in the *O. biroi* genome, has characteristics that suggest recombination was suppressed in the ancestral sexual population: a heteromorphic karyotype, at least one large inversion, an enrichment of structural variants and TEs, and a proportional reduction of exons (Figure 6, C–G). Second, the loss of heterozygosity breakpoints do not appear to have affected any protein-coding genes (Data S3D), and all of the variants we identified on Chromosome 13 are losses of heterozygosity, suggesting that the phenotype did not result from a *de novo* variant on Chromosome 13 in QLMs. Finally, Chromosome 13 contains a large array of *CYP9* genes that have many sequence differences between haplotypes (Figure 7A) and may be involved in hormone signaling. However, we cannot definitively prove the supergene hypothesis with the available data, and it is in principle possible that the QLMs were produced via mutations elsewhere in the genome that arose during the clonal evolution of Line A.

Regardless of the specific genetic cause, the QLMs demonstrate that a modular genetic architecture can allow an obligate workerless social parasite to emerge within its parental colony and persist for a period of time. This unexpected finding finally provides an empirical solution to the puzzle of workerless social parasite evolution that is consistent with molecular phylogenetic evidence on the evolution of inquilines and growing recognition that supergenes regulate many types of social polymorphisms^14,15,19,24–26,62^.

## STAR Methods

### RESOURCE AVAILABILITY

#### Lead contact

Further information and requests for resources and reagents should be directed to and will be fulfilled by the lead contact, Waring Trible (bucktrible@g.harvard.edu).

#### Materials availability

This study did not generate new unique reagents. All biological materials other than ants are commercially available, and ants can be provided on request, in accordance with federal regulations.

#### Data and code availability

- Raw morphometric data are available in Data S2.
- Raw Illumina sequencing reads are available at the National Center for Biotechnology Information Short Reads Archive, BioProject PRJNA923657.
- *De novo* genome assemblies (Obir v5.6, Obir v5.7, WT Flye, and QLM Flye) are available at https://github.com/triblelab/qlm.
- Original code is available at https://github.com/triblelab/qlm.
- Any additional information required to reanalyze the data reported in this paper is available from the lead contact upon request.

### EXPERIMENTAL MODEL AND SUBJECT DETAILS

*Ooceraea biroi* colonies were reared as reported previously^65^. Briefly, colonies were maintained at 25°C in circular Petri dishes (50 mm diameter, 9 mm height) with a plaster of Paris floor ca. 4 mm thick. Colonies were fed 3 times weekly with fire ant (*Solenopsis invicta*) brood and cleaned and watered at least once per week. In the context of this manuscript, wild-type (WT) always refers to phenotypically normal ants derived from Clonal Line A of *O. biroi*^28^. For penetrance and larval survival, colonies of 100% WT or 100% QLM adults (assessed by phenotype) were established without brood. Eggs were removed, allowed to hatch into larvae on glass slides, and then fostered into units to be reared by 16 WT or 16 QLM adults (also referred to as chaperones).

### METHOD DETAILS

#### Microsatellite and COI sequencing

PCR amplification of *cytochrome oxidase I* (COI) was performed using primers as reported previously^28^. Amplification was performed in a final volume of 12 μL using Applied Biosystems’ AmpliTaq Gold Kit following manufacturers’ recommendations. Cycling profiles started with a denaturation at 94°C for 10 min, and then proceeded with 40 cycles of 94°C for 30 s, 55°C annealing for 30s, 72°C extension for 30 s, finally followed by an extension of 72°C for 10 min. Microsatellite reagents and cycling profiles followed a previous report^28^. Fragment analysis was performed by GeneWiz (Frederick, MD). Microsatellite loci were scored using Peak Scanner (Applied Biosystems).

#### Morphometrics measurements

Individual ants were dissected to count ovarioles, and then mounted and imaged. Linear measurements were taken using the Fiji distribution of ImageJ^66^. Fifteen measures were used for morphometric analysis, extracted from side view, top view, and head view images. Side view measures were: eye length, head length, head height, Weber’s length, mesosoma height, and length of 1^st^ gastral segment (Figure S1). Top view measures were: mesosoma length, mesosoma width, mesosoma segment number, petiole length, petiole width, postpetiole length, and postpetiole width. Head view measures were: head length, head width. To produce a size-matched dataset, we excluded the largest QLMs and smallest WTs until the means of the population were not significantly different, resulting in exclusion of the 7 smallest WTs and 8 largest QLMs. All morphometric analyses were performed and reported using both the full dataset and the size-matched dataset.

Principal components analysis was conducted in Prism 7 using a normalized dataset that combines our measurements with published measurements of *O. octoantenna* and *O. siamensis* ^1,2^. To enable cross-species comparisons, normalization was achieved by linearly scaling each trait in each species to range from zero to one (Data S2C).

#### Foraging behavior

For behavior and fitness datasets, particular care was taken to specifically measure the effect of genotype (QLM versus WT) while controlling for age and rearing environment. Colonies used for experimental treatments were always matched to control for cohort variation among the chaperone ants.

Foraging experiments were conducted as reported previously^39^. Briefly, ants were maintained in a two-part dish with a 2cm diameter nest connected by a narrow opening to a 6.5cm diameter foraging arena. Ants were placed in the nest (with the opening to the foraging arena sealed) without brood and allowed to lay eggs. Once these eggs hatched into larvae (approximately 14 days later), the seal was removed to allow foragers to leave the nest, and food was placed in the foraging arena. Videos of foraging behavior and subsequent food retrieval were recorded and manually annotated as reported previously^39^. Colonies were fed, watered, and used for behavioral recordings every day until the larvae entered the prepupal stage (approximately 14 days after initiating recordings). This yielded approximately 10–15 foraging raids per colony. Small numbers of ants that died during the course of the experiment were removed the following day.

All behavioral experiments consisted of colonies established using 16 ants that were painted with color tags to make it easier to identify their genotype in the video recordings. Ant colonies were established using a strict paired design, such that every group of QLM ants was paired to a group of WT ants that was produced by the same rearing unit and subsequently reared in an identical manner. Ants ranged from 14 days to 2 months old at the start of experiments, and ages were matched within each pair. Pure colonies were all QLM (n = 4) or WT (n = 4). Mixed colonies (n = 8) were established with 8 QLMs and 8 WTs.

#### Fitness measurements

Egg-laying rates were measured from five replicate QLM and WT colonies that were matched for age, colony size, and rearing environment. Egg to pupa survival and eclosion survival were measured by establishing colonies with WT chaperones and varying proportions of QLM and WT brood.

Efficiency of larval rearing was measured using colonies with 20 WT or QLM chaperones established with <24h old eggs. When these eggs hatched into larvae, colonies were reduced to 16 first-instar larvae. Developmental transitions were scored as the date that the majority of surviving individuals had entered the respective developmental stage (1^st^ instar, 2^nd^ instar, 3^rd^ instar, 4^th^ instar, prepupal stage, pupal stage, and eclosion). Overall survival from larva to adult was scored at the end of the experiment.

#### Whole-genome sequencing (Illumina)

17 DNA libraries were used for whole-genome Illumina sequencing. Tissues from individual adult ants were disrupted in a tissue lyser and genomic DNA was extracted using Qiagen’s QIAmp DNA Micro Kit following manufacturer’s recommendations. Libraries were prepared using Nextera Flex. Paired-end 150 base pair reads were sequenced on an Illumina NovaSeq SP Flow Cell. We aimed for 35X coverage to ensure sufficient read depth to accurately detect heterozygosity across the genome (Data S3A).

Short read data were processed following the GATK best practices pipeline^69^ using the Obir v5.6 genome assembly generated in this study. Briefly, raw reads were trimmed with Trimmomatic 0.36^67^, aligned using BWA-MEM^68^, and sorted and de-duplicated before variants were called using HaplotypeCaller^69^.

Read depth distributions can contain excessively high and low values due to errors in genome assembly. These values can augment or diminish estimates of mean read depth across genomic regions. To avoid these effects, read depth was calculated using samtools depth^70^ at stringently filtered SNPs. In addition to the standard GATK-recommended filtration steps, SNPs with null genotype calls, and SNPs with one or more samples having read depth greater than or equal to 80 (double the median read depth) were removed from the dataset. Because read depth differs among samples due to minor differences in library concentration during loading onto the Illumina flow cell, the normalized read depth was calculated by dividing the read depth at each SNP by the median read depth across all SNPs. Next, the mean normalized read depth was calculated for SNPs within each contig.

For molecular phylogenetics, whole-genome SNP calls (excluding sites with missing data in any library) were converted from vcf to fasta using vcf2phylip (https://github.com/edgardomortiz/vcf2phylip), such that heterozygous sites were represented using IUPAC uncertainty codes. These files were then used to create a diploid-aware phylogeny with the -GENOTYPE setting in RAxML-NG^71,72^. RAxML^72^ was then used to estimate bootstrap support using the model -GTGTR4+G with 100 replicates.

Sites that distinguished the QLMs were defined as sites where both QLM libraries had the same genotype and differed from all 15 WT libraries. These sites were identified using a custom R script (https://github.com/triblelab/qlm).

#### Improved genome assembly

To improve the assembly of Chromosome 13, we re-analyzed published data using the Falcon pipeline. First, we ran Falcon-unzip^73^ using published PacBio data^45^, yielding primary (212Mbp, 322 contigs with N50 of 2.2Mbp) and alternate (67.8Mbp, 2685 contigs with N50 of 27.37kbp) assemblies. These assemblies were phased with published Hi-C data^45^ using Falcon-phase^74^, and then scaffolded using the Obir v5.4 genome assembly using RaGOO^75^. Finally, this primary assembly, which we refer to as the Obir v5.6 assembly, was polished and masked as described previously^45^.

Next, we performed high molecular weight extractions of *O. biroi* following a published protocol^76^ and sequenced two barcoded libraries using the Oxford Nanopore PromethION platform. Raw reads were assembled *de novo* using Flye (version 2.8.2)^77^ and aligned to the Obir v5.6 contigs using Mauve (snapshot 2015–02-25)^78^. This allowed us to manually re-order and re-orient the contigs of v5.6 by aligning them with the contigs of the two *de novo* assemblies as well as the Obir v5.4 assembly, yielding an improved Obir v5.7 assembly. Assemblies v5.6 and v5.7 do not differ in sequence content, but only in the position and orientation of Chromosome 13 contigs (see Data S3C).

#### Characterization of Chromosome 13

To document transposable element (TE) content in *O. biroi* and other species, we used RepeatModeler and RepeatMasker (Smit, AFA, Hubley, R & Green, P., repeatmasker.org) to identify TEs belonging to known transposon classes from published genome assemblies (*O. biroi*: GCA_003672135.1; *S. invicta*: GCA_010367695.1; *D. melanogaster*: GCA_000001215.4). To quantify gene content, we used the entire set of exons from the NCBI annotation of Obir v5.4 as a proxy (https://www.ncbi.nlm.nih.gov/genome/annotation_euk/Ooceraea_biroi/100/). We excluded predicted TE sequences that overlapped with annotated exons and exonic sequences that overlapped with predicted TEs from the analyses. We calculated the proportion of each scaffold comprised of each feature as the number of base pairs occupied by predicted TEs or annotated exons divided by the ungapped length of each scaffold in base pairs.

#### Gene-level analysis

To identify genes in the loss of heterozygosity region, we used the Mauve alignments (above) to identify contigs in the v5.4 genome assembly that aligned to the loss of heterozygosity region. We then used the published NCBI *O. biroi* annotation v5.4^45^ to identify genes within those contigs. Genes in the loss of heterozygosity region were then tested for GO and KEGG enrichment using a custom R script (https://github.com/triblelab/qlm).

*O. biroi CYP9* genes were initially identified using a BLAST search using an exemplar protein (*O. biroi CYP9_1_1*). These results were then curated using the NCBI genome browser and amino acid sequence alignments to ensure that each gene name refers to a single enzyme. Every gene we report here is transcribed and contains intact CYP functional domains, implying that they are not pseudogenes. However, some *CYP9* genes lack start codons and may be transcribed and translated with their 5’ neighbor. The spatial array of *CYP9* genes was created in Adobe Illustrator using start and stop coordinates of each exon. The phylogeny of *CYP9* genes was generated using RAxML. This phylogeny was also used to identify and remove proteins annotated as *CYP9s* that actually belong to the *CYP6* subfamily.

To quantify *CYP9* conservation and expansion, we obtained published CYP9 amino acid sequences from *Apis mellifera* and *Nasonia vitripennis*^52^, and the procedure described above was repeated for CYP9 proteins in three additional ant species that possess high-quality genomes and span the ant phylogeny (NCBI: *Harpegnathos saltator* Hsal v8.5, *Monomorium pharaonis* ASM326058v2, and *Camponotus floridanus* Cflo v7.5). Ant CYP9 enzymes were identified using a BLAST search with *O. biroi* CYP9_1_1 against the genome of each of the four ant species. All identified sequences were downloaded, and duplicates and sequences that fall phylogenetically outside of the insect CYP9 clade (as defined previously^52^) were removed. Note that 12 *CYP9* genes occur in the loss of heterozygosity region according to the Obir v5.4 genome assembly and annotation, but manual re-annotation of this assembly identified 24 *CYP9* genes in this region (Data S3F). Amino acid sequences are available at https://github.com/triblelab/qlm.

### QUANTIFICATION AND STATISTICAL ANALYSIS

Statistical analyses were performed using Graphpad’s Prism 7, unless otherwise stated, and the details of statistical tests can be found in the figure legends. Code and packages used for genomics analyses are reported in **DATA AND SOFTWARE AVAILABILITY** and **METHOD DETAILS**.

## Supplementary Material

1 Data S1: Definitions of caste terminology and ant caste evolution, related to Figure 1.

2 **Data S2: Raw morphometric dataset and principal component values, related to** Figures 2 and 3. (A) Full O. biroi morphometric dataset. (B) Raw combined data from O. biroi, O. octoantenna, and O. siamensis. (C) Normalized combined data and principal component scores.

3 **Data S3: Supporting information for genomic analyses, related to** Figures 6 and 7. (A) Coverage per base pair for whole-genome sequencing. Sequencing libraries from 17 individual ants were aligned to the Obir v5.4 genome assembly (1st quartile, median, 3rd quartile, mean). Average coverage was approximately 35X. (B) Polymorphisms outside of the loss of heterozygosity region that are unique to QLMs. Heterozygous sites unique to QLMs were defined as gain-of-heterozygosity and correspond to point mutations. Short homozygous regions identified here (up to 1kbp interval) were defined as loss of heterozygosity. Unlike the large loss of heterozygosity on Chromosome 13, these small homozygous intervals likely correspond to gene conversion events. Following these criteria, the 36 SNPs unique to QLMs correspond to 28 separate mutations: 9 gain of heterozygosity and 19 loss of heterozygosity eventsS21,S22. Four mutations affect exons, but three of these are synonymous substitutions and the fourth is not in a functional domain. 12 mutations are non-coding (intronic or <1kbp from an exon), and the remaining 12 mutations are intergenic (>1kbp from an exon). A small number of neutral mutations are expected to arise during clonal evolution and, unlike the large loss of heterozygosity on Chromosome 13, none of the genes associated with these mutations are a priori candidates for caste differentiation. (C) Evidence used to produce a contiguous ‘Obir v5.7’ assembly of Chromosome 13. First, the C1 WT and QLM de novo assemblies were aligned to the v5.6 assembly, and contigs from either de novo assembly that join contigs in the v5.6 assembly are listed (second and third columns). Chromosome 13 contigs in the v5.6 assembly were scaffolded by alignment to the Hi-C-scaffolded v5.4 assembly using RaGOO, so contigs at adjacent positions in the v5.6 assembly indicate that the contig gap was supported by a v5.4 contig and/or Hi-C data (fourth column). This allowed us merge and re-orient the 54 contigs of the Obir v5.6 genome assembly (polished output of Falcon-phase and RaGOO), yielding the Obir v5.7 assembly. (D) Start and stop positions of the loss of heterozygosity in QLMs. Loss of heterozygosity start and stop positions were defined as the position at which QLM genotype calls transition between heterozygous and homozygous genotypes in the contiguous Obir v5.7 assembly. To identify potential genes affected by the loss of heterozygosity, the homologous positions in the v5.4 assembly were identified using BLAST. (E) List of heterozygous structural variants within the loss of heterozygosity region. Structural variants were inferred using a comparison of the primary and secondary Chromosome 13 contigs from C1 WTs. The loss of heterozygosity in QLMs spans 81 structural variants that encompass 98,208 bp. This indicates that C1 WTs are heterozygous for two highly divergent haplotypes at these loci, and the QLMs have become homozygous for one of the two haplotypes. (F) List of annotated genes within the loss of heterozygosity region, with associated variants affected by loss of heterozygosity. Variants are defined as in Data S3A. In the v5.4 assembly, the loss of heterozygosity contains 188 genes, and these are associated with 70 variants. Re-annotations are provided for the 24 CYP9 genes (annotated as 17 genes in the NCBI 5.4 genome version) in the loss of heterozygosity region. (G) GO term enrichment of genes within the loss of heterozygosity region. (H) KEGG ortholog enrichment of genes within the loss of heterozygosity region.

4

5 **Video S1: Exemplar WT foraging raid, related to** Figure 4. Ten WTs participated in the raid at its peak.

6 **Video S2: Exemplar QLM foraging raid, related to** Figure 4. Two QLMs participated in the raid at its peak.

## Figures and Tables

**Key resources table:** 

REAGENT or RESOURCE	SOURCE	IDENTIFIER
**Critical commercial assays**		
QIAamp DNA Micro Kit	QIAGEN	56304
Amplitaq Gold	Applied Biosystems	4398881
Nextera Flex Library Preparation Kit	Illumina	20060059
NovaSeq SP Flow Cell	Illumina	20027464
Ligation Sequencing Kit	Oxford Nanopore	SQK-LSK109
PromethION Flow Cell	Oxford Nanopore	FLO-PRO002
**Deposited data**		
*De novo* assemblies & code	This paper	https://github.com/triblelab/qlm
Raw Illumina sequencing reads	This paper	
**Experimental models: Organisms/strains**		
*Ooceraea biroi* Clonal Line A	Kronauer et al. 2012^28^	C1, C2, C5, C13, C16, and C17
*Ooceraea biroi* queen-like mutant	This paper	N/A
**Oligonucleotides**		
Microsatellite primers	Kronauer *et al.* 2012^28^, Butler et al. 2014^79^	DK371, ES177, D8Z0W, D8M16, D4XW2, ER4IH
LCO: 5’- GGTCAACAAATCATAAAGATATTGG-3’	Folmer *et al.*, 1994^80^	IDT: LCO
HCO: 5’-TAAACTTCAGGGTGACCAAAAAATCA-3’	Folmer *et al.*, 1994^80^	IDT: HCO
**Software and algorithms**		
PeakScanner	Applied Biosystems	v1.0
ImageJ	Schneider et al.^81^	https://imagej.nih.gov/ij/download.html
GATK	Van der Auwera et al.^82^	https://gatk.broadinstitute.org/hc/en-us
samtools	Li et al.^70^	http://www.htslib.org/download/
Trimmomatic v 0.36	Bolger et al.^67^	https://github.com/usadellab/Trimmomatic
RAxML	Stamatakis^71,72^	https://github.com/stamatak/standard-RAxML
Python3	Python Software foundation	https://www.python.org/psf/
GraphPad Prism	GraphPad Prism	Prism v7
R	R Core Team	http://www.R-project.org/
GO+KEGG.Rmd	This paper	N/A
qlm_LOH.Rmd	This paper	N/A
Falcon-unzip	Chin et al.^73^	https://pb-falcon.readthedocs.io/en/latest/about.html
Falcon-phase	Kronenberg et al.^74^	https://github.com/phasegenomics/FALCON-Phase
RaGOO	Alonge et al.^75^	https://github.com/malonge/RaGOO
Mauve (snapshot 2015-02-25)	Darling et al.^83^; Rissman et al.^78^	http://darlinglab.org/mauve/
RepeatModeler	Smit, AFA, Hubley, R. Unpublished	http://www.repeatmasker.org/
RepeatMasker	Smit, AFA, Hubley, R & Green, P. Unpublished	http://www.repeatmasker.org/
topGO	R package, Alexa and Rahnenfuhrer	https://bioconductor.org/packages/release/bioc/html/topGO.html
eggnog-mapper	Huerta-Cepas^84^	http://eggnog5.embl.de
Kyoto Encyclopedia of Genes and Genomes (KEGG)	KAAS: an automatic genome annotation and pathway reconstruction server	https://www.genome.jp/kegg/
Prism 7	Graphpad Software	https://www.graphpad.com/scientific-software/prism/

## Highlights

- A mutant ant strain resembles known social parasites without a worker caste
- These parasitic queen-like variants arose directly from a free-living ancestor
- The variants have a mutant supergene that probably causes their queen-like traits
- This suggests that supergenes can underlie the evolution of ant social parasitism
