# Supplementary material for: A caste differentiation mutant elucidates the evolution of ant social parasites
Source: Curr Biol. Author manuscript; Available in PMC 2023 Mar 29. (PMC10050096; doi:10.1016/j.cub.2023.01.067)
Supplement: 1 — Data S1: Definitions of caste terminology and ant caste evolution, related to Figure 1. [file NIHMS1873106-supplement-1.doc]

Data S1: Definitions of caste terminology

Castes can be classified using three mechanistically distinct categories: physiology, behavior, and morphology. All discussions of caste development in this manuscript refer to caste morphology, and our use of the term ‘caste’ generally refers to caste morphology unless otherwise noted. We use the term ‘queen’ exclusively to refer to female adults with a suite of queen-like morphological traits (wings, ovaries, and eyes), and ‘worker’ to refers to adults with worker-like morphological traits (wingless, reduced ovaries and eyes)^S24^.

We describe **caste physiology** using the terms ‘reproductive’, for individuals whose ovaries are active or have the potential to activate, and ‘non-reproductive’, for individuals whose ovaries are inactive or lack the potential to activate. Workers of *O. biroi*, as with other queenless, clonal social insects, are reproductive, but they are not queens in the morphological sense: *O. biroi* workers are morphologically normal and comparable to workers of related *Ooceraea* species that retain winged queens (Figure S4). There is no evidence that the evolution of parthenogenesis (which acts on the mechanisms of meiosis in gametes) in *O. biroi* involved a change to caste differentiation (which acts on the mechanisms of metamorphosis in late larvae). For instance, the cape honeybee is a genotypic variant that has also evolved the capacity for morphologically normal workers to reproduce via thelytokous parthenogenesis, but this genotype retains the ability to produce morphologically normal queens^S25^. Finally, the QLMs reproduce parthenogenetically, and the QLMs are morphologically unlike the ancestral winged queens of *O. biroi*. The QLMs therefore do not represent revertants to the ancestral sexual phenotype, or atavisms.

We describe **caste behavior** as either worker-like or queen-like, with foraging representing an example of worker-like behavior. It is important to note that caste behavior and caste morphology are not invariably associated; queens of many species can exhibit worker-like behavior under certain contexts (such as during an early phase of the life cycle), and experimental manipulations can decouple caste morphology from caste behavior^S26,S27^. The conclusion that the QLMs display an induction of queen-like morphology does not necessarily imply that they would also exhibit a reduction in worker-like behavior.

**Caste morphology** is measured in female adults and is irreversibly attained during metamorphosis, and the program of larval growth that precedes metamorphosis is broadly defined as caste development. **Caste development** consists of at least two major steps: **caste determination**, which regulates the probability that an egg or larva will develop into a particular caste, and **caste differentiation**, which regulates the caste-specific growth of tissues. Within a genetic background, caste morphology varies with body size: larger individuals metamorphose with more queen-like features, and this morphological variation can therefore be described using a **caste reaction norm**, a continuum from small female adults with worker-like morphology to large female adults with queen-like morphology (Figure S2)^S6^.

Based on existing evidence, genetic and experimental perturbations that affect caste determination alter adult caste morphology if and only if they also give rise to corresponding differences in adult body size^S6,S7^. Developmental or genetic perturbations that affect **caste determination** thus result in changes both in body size and caste morphology, but do not alter the relationship between size and morphology or the relationship between caste-associated traits in comparisons of equally-sized individuals. For instance, a genotype with a genetic bias for queen determination will display morphologically normal queens that are indistinguishable from those produced via phenotypic plasticity. Rare small individuals, if they are ever observed, display typical worker morphology, rather than developing into worker-sized queens^S6,S28,S29^. These statements are also true for lineages with wingless queens (i.e., morphological queens below the threshold size for wing development) and microgynes (i.e., morphological queens below the size of regular queens but above the threshold size for wing development)^S6^. These results imply that these phenotypes arise via changes affecting caste determination (Figure S2).

In contrast, perturbations affecting **caste differentiation** result in changes to the coordination of growth between tissues, and/or an atypical association of body size and caste morphology. A genotype with a genetic bias for queen differentiation is expected to produce queens that differ morphologically from typical queens, and so changes to body size should not have the capacity to rescue typical worker development in such genotypes; rare small individuals are expected to retain queen-like tissue growth.
