# Supplementary material for: A caste differentiation mutant elucidates the evolution of ant social parasites
Source: Curr Biol. Author manuscript; Available in PMC 2023 Mar 29. (PMC10050096; doi:10.1016/j.cub.2023.01.067)
Supplement: 4 [file NIHMS1873106-supplement-4.pdf]

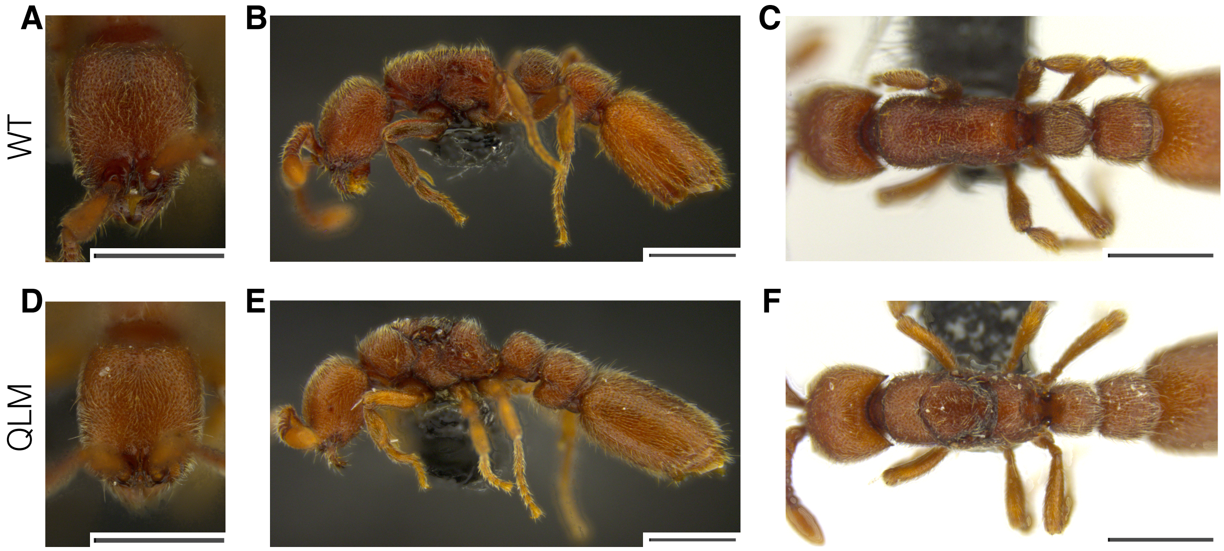

Figure S1. WT and QLM images, related to Figures 2 and 3. (A) and (D) WT and QLM head frontal view. (B) and (E) WT and QLM side view. Gaster tips were removed for ovariole dissections. (C) and (F) WT and QLM top view. Scale bars in (A-F): 0.5mm.

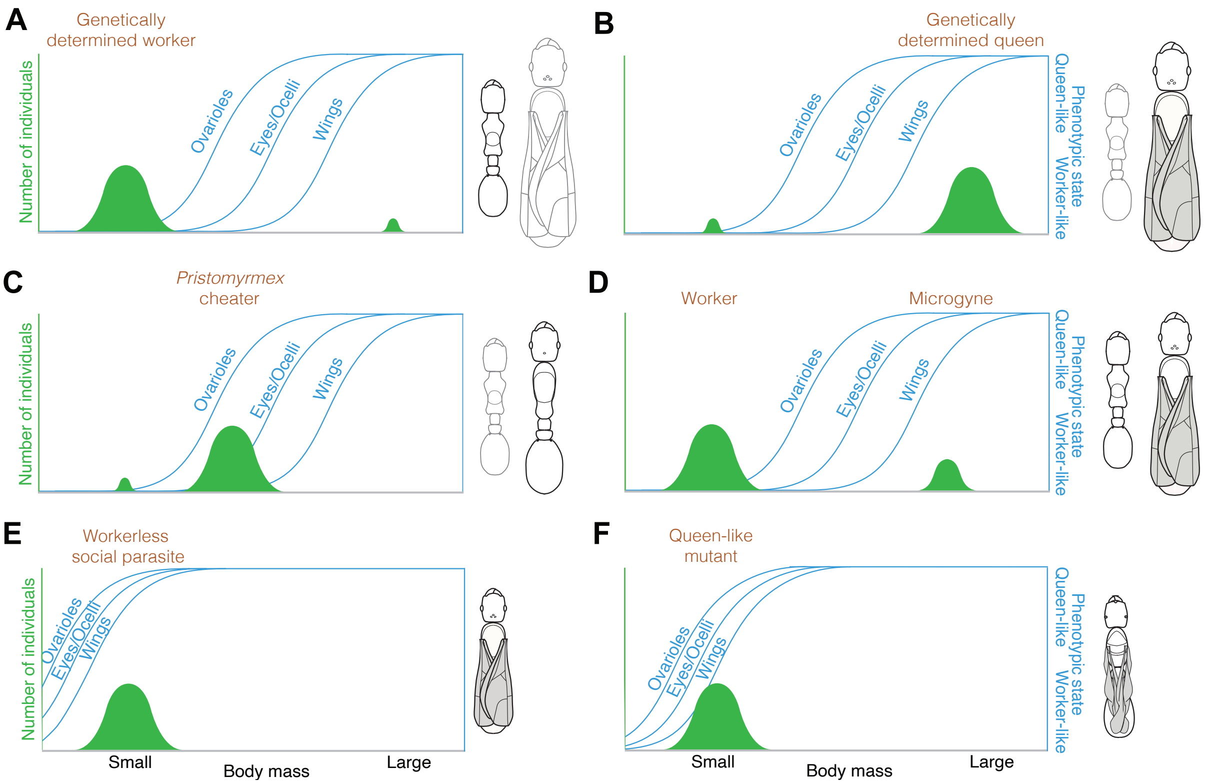

Figure S2. Cartoons and caste reaction norms for genetic variation in ant caste development, related to Figures 2 and 3. (A) Genetic bias for worker determination. Genetic biases for worker development are associated with thelytokous parthenogenesis of reproductive workers, as in *Ooceraea biroi* and *Pristomyrmex punctata*. Species with social hybridogenesis, such as *Wasmannia auropunctata* and *Cataglyphis mauritanica*, display genetic biases for worker development in certain genotypes and queen development in other genotypes[S1–S5]. These genetic systems act by increasing the probability that a larva will develop into a worker (potentially up to 100%) and can be interpreted as a genetic bias to body size that indirectly affects caste morphology[S2,S6,S7]. There is no evidence that genetic caste determination systems alter the mechanisms of caste differentiation or allometric scaling. *O. biroi* workers are morphologically comparable to those of related species (Figure 3) and, while winged queens have never been observed, large individuals have partially queen-like traits, indicating that the clonal raider ant displays a genetic bias for worker determination[S8]. (B) Genetic biases for queen determination are primarily documented from species with social hybridogenesis, which display genetic biases for worker development in certain genotypes and queen development in other genotypes. Most workerless social parasites display miniaturized queens that differ in allometric scaling relative to their closest free-living relatives (see panel E), but some large-bodied workerless social parasites may effectively represent genetically determined queens[S9–S11]. (C) Reproductive cheater genotype of *Pristomyrmex punctata*[S2]. Like in *O. biroi*, most clonal lines of *Pristomyrmex punctata* display a genetic bias for worker determination (A), but this divergent lineage shows a wingless queen phenotype: an increase in body size with partially queen-like ovaries, eyes, and mesosoma. Multiple free-living sexual *Pristomyrmex* species possess wingless queens with the same morphological syndrome as these cheaters, and rare small individuals of this cheater genotype display typical worker morphology, so we can conclude that this phenotype arose via a genetic bias toward larger body size, analogous to (B)[S12,S13]. Furthermore, these

cheaters do not morphologically resemble workerless social parasites and, unlike the *O. biroi* QLMs, they belong to their own clonal line, so it is not possible to infer the genetic architecture for social parasitism in this case. (D) Microgynes. Many facultatively parasitic species retain phenotypic plasticity for worker and queen development but have evolved microgynes, i.e., queens that are smaller than the typical queens of their genus. Unlike QLMs and workerless social parasites, microgynes appear to be equal to or larger in size than workers[S7,S14–S16]. Therefore, the evolution of microgynes represents a reduction in the size dimorphism between workers and queens, but there is no evidence that it entails an induction of queen-like morphology at worker-like body size. Recent phylogenetic and comparative evidence has failed to support the hypothesis that species with facultatively parasitic microgynes – or other forms of facultative social parasites – represent the evolutionary intermediates between free-living ants and workerless social parasites[S9,S17]. Instead, the closest relatives of most workerless social parasites appear to be free-living ants with phenotypically normal queens. (E) Workerless species with worker-sized queens. Every described species with this combination of morphological traits is an obligate inquiline (i.e., a workerless social parasite). Some of these workerless social parasites can produce rare worker offspring but, unlike in (B-D), these workers are morphologically abnormal and have extremely small body size[S18–S20]. Therefore, these workerless social parasites display a genetic change to the allometric scaling mechanisms of caste differentiation[S6]. This observation implies that, as in *O. biroi* QLMs, genetic lineages with worker-sized queens have workers that are reduced or absent. The only documented exception to this trend is the *Formica difficilis* species group, which has worker-sized queens and retains regular workers[S14,S17]. (F) *O. biroi* QLMs. To our knowledge, the QLMs represent the only documented intraspecific variant that lacks workers and displays queen-like morphology at worker-like body size in an ant. Like workerless social parasites (E), the QLMs display a change to allometric scaling. No such change has been demonstrated for genetically determined queens (B), *Pristomyrmex* cheaters (C), or microgynes (D). Note that, relative to typical *Ooceraea* queens (Figure 3), the morphology of the QLMs is uncoordinated: the wings do not inflate following eclosion and QLMs have only partially queen-like eyes and ocelli. This unusual combination of morphological features provides further evidence that the QLMs display a radical alteration to the allometric scaling mechanisms of caste differentiation.

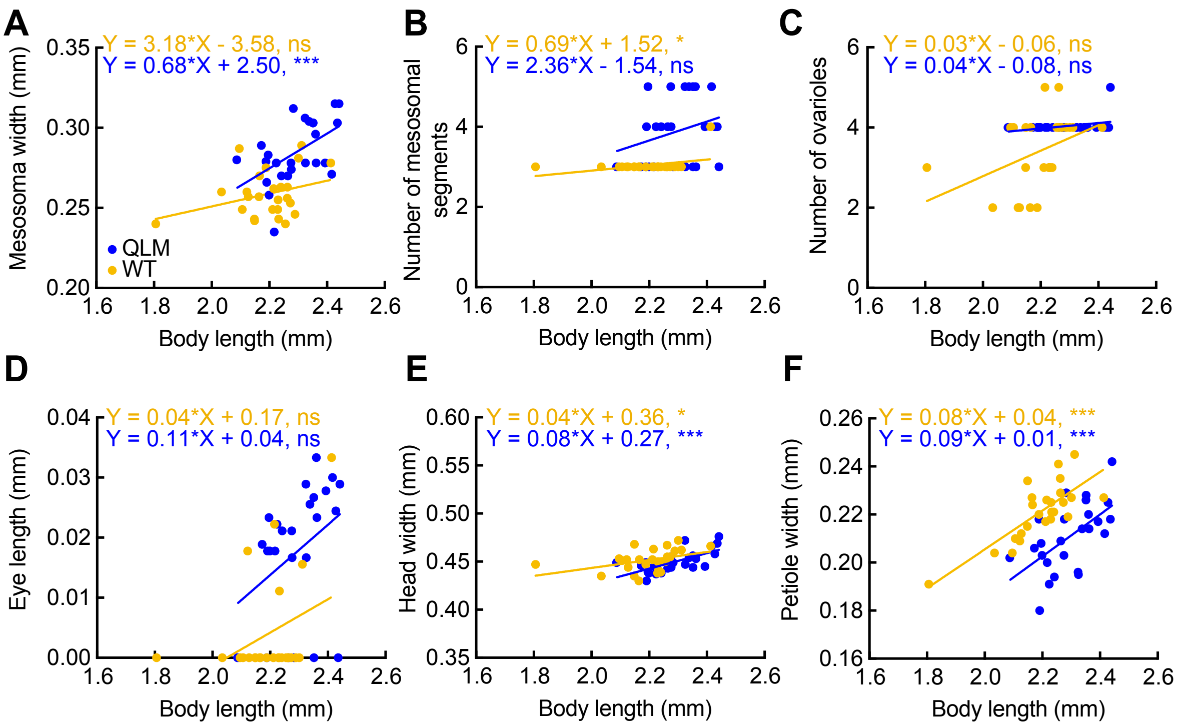

Figure S3. Supplementary WT and QLM morphometrics, related to Figures 2 and 3. (A) Mesosomal width. (B) Number of mesosomal segments. Three segments are characteristic of worker ants, while five segments are characteristic of queen ants. (C) Number of ovarioles. (D) Maximal length of pigmented photoreceptor patch of the eye. Values of 0 indicate none visible. (E) Head width. (F) Petiole width. All graphs depict n = 25 WT and QLM adults. ns: not significant; $*p<0.05$; $***p<0.001$. p values from linear regression (Dfd = 23) with an expected slope of zero. Equations provide best-fit slope and y-intercept.

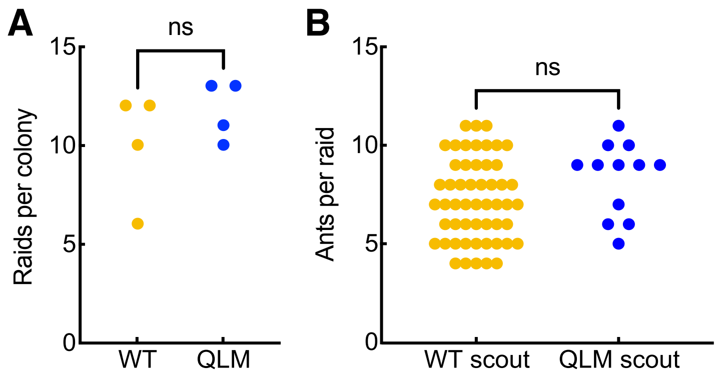

Figure S4. **Equal number and size of raids in WTs and QLMs, related to Figure 4.** (A) Raids per colony in 100% WT (n = 4) and 100% QLM (n = 4) colonies. QLM colonies do not engage in fewer raids than WT colonies. (B) Ants participating in raids initiated by WT or QLM scouts in mixed colonies (n = 66 raids). QLM scouts do not recruit fewer ants per raid than WT scouts.

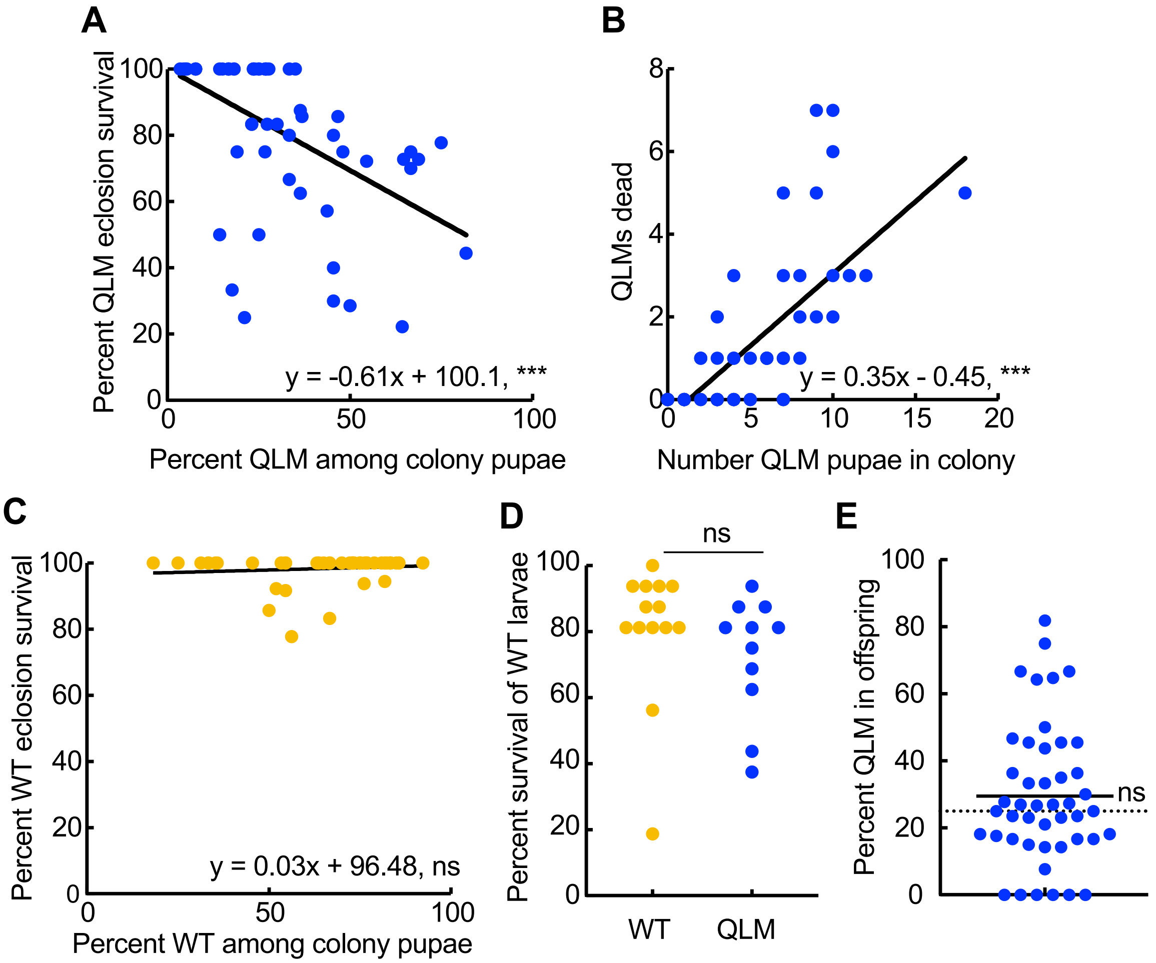

Figure S5. **QLMs show frequency-dependent survival as pupae, but rear larvae efficiently and persist at stable frequencies, related to Figure 5.** (A) Survival during eclosion for QLM pupae as a function of the percent of QLM pupae within the colony. (B) Number of QLM pupae that died during eclosion as a function of the number of QLM pupae within the colony. (C) Survival of WT pupae as a function of the percent of WT pupae within the colony. (D) Percent survival of WT larvae that were reared by WT or QLM adults. (E) Percent of QLM adults produced by colonies with 5 QLM and 15 WT adults. Percent of QLM adults produced was not significantly different from the percent of QLM adults in the colony (25%), implying that the frequency of QLM adults can remain approximately stable across generations. p values in (A-C) from linear regression with an expected slope of zero. Equations provide best-fit slope and y-intercept. p value in D from unpaired t-test, and p value in E from one-way Wilcoxon test. ns: not significant, ***$p<0.001$.

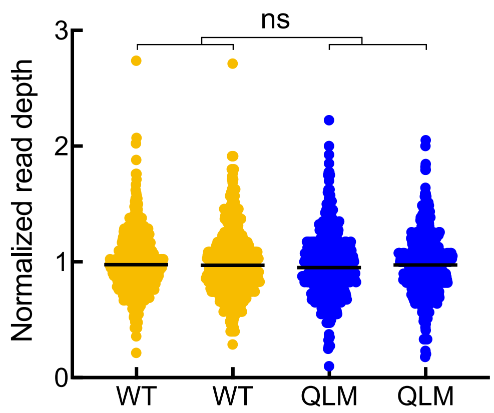

Figure S6. **Normalized read depth at Chromosome 13 loci, related to Figure 6.** QLMs do not differ from WTs in read depth at the loss of heterozygosity loci on Chromosome 13, indicating that the homozygous interval on Chromosome 13 represents a loss of heterozygosity rather than a deletion. ns: not significant. p value from paired t-test comparing WT and QLM average at each locus.

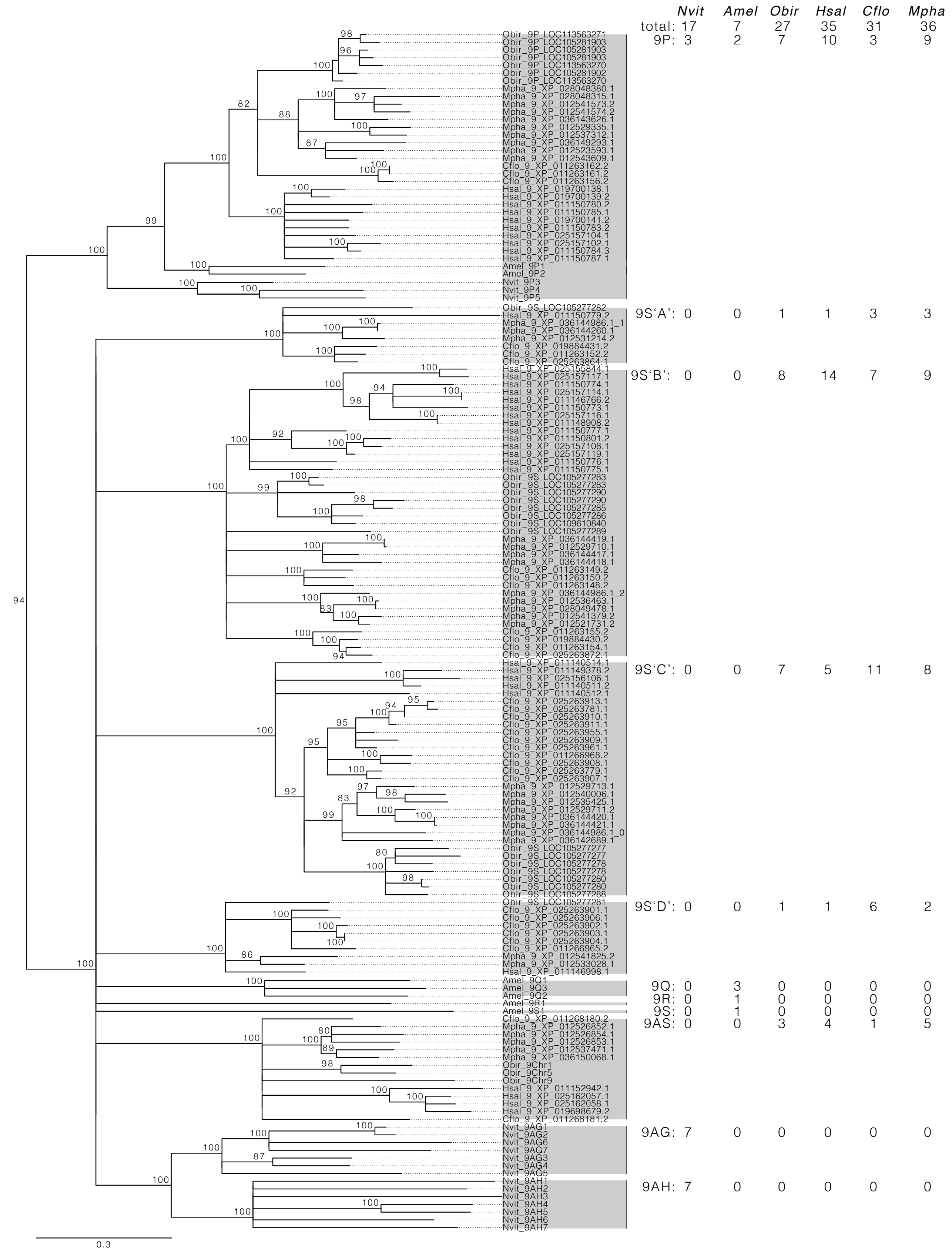

Nvit Amel Obir Hsal Cflo Mpha
total: 17 7 27 35 31 36
9P: 3 2 7 10 3 9
9S'A': 0 0 1 1 3 3
9S'B': 0 0 8 14 7 9
9S'C': 0 0 7 5 11 8
9S'D': 0 0 1 1 6 2
9Q: 0 3 0 0 0 0
9R: 0 1 0 0 0 0
9S: 0 1 0 0 0 0
9AS: 0 0 3 4 1 5
9AG: 7 0 0 0 0 0
9AH: 7 0 0 0 0 0
Obir_9P_LOC113563271
Obir_9P_LOC105281903
Obir_9P_LOC105281903
Obir_9P_LOC105281903
Obir_9P_LOC113563270
Obir_9P_LOC105281902
Obir_9P_LOC113563270
Mpha_9_XP_028048380.1
Mpha_9_XP_028048315.1
Mpha_9_XP_012541573.2
Mpha_9_XP_012541574.2
Mpha_9_XP_036143626.1
Mpha_9_XP_012529335.1
Mpha_9_XP_012537312.1
Mpha_9_XP_036149293.1
Mpha_9_XP_012523593.1
Mpha_9_XP_012543609.1
Cflo_9_XP_011263162.2
Cflo_9_XP_011263161.2
Cflo_9_XP_011263156.2
Hsal_9_XP_019700138.1
Hsal_9_XP_019700139.2
Hsal_9_XP_011150780.2
Hsal_9_XP_011150785.1
Hsal_9_XP_019700141.2
Hsal_9_XP_011150783.2
Hsal_9_XP_025157104.1
Hsal_9_XP_025157102.1
Hsal_9_XP_011150784.3
Hsal_9_XP_011150787.1
Amel_9P1
Amel_9P2
Nvit_9P3
Nvit_9P4
Nvit_9P5
Obir_9S_LOC105277282
Hsal_9_XP_011150779.2
Mpha_9_XP_036144986.1_1
Mpha_9_XP_036144260.1
Mpha_9_XP_012531214.2
Cflo_9_XP_019884431.2
Cflo_9_XP_011263152.2
Cflo_9_XP_025263864.1
Hsal_9_XP_025155844.1
Hsal_9_XP_025157117.1
Hsal_9_XP_011150774.1
Hsal_9_XP_025157114.1
Hsal_9_XP_011146766.2
Hsal_9_XP_011150773.1
Hsal_9_XP_025157116.1
Hsal_9_XP_011148908.2
Hsal_9_XP_011150777.1
Hsal_9_XP_011150801.2
Hsal_9_XP_025157108.1
Hsal_9_XP_025157119.1
Hsal_9_XP_011150776.1
Hsal_9_XP_011150775.1
Obir_9S_LOC105277283
Obir_9S_LOC105277283
Obir_9S_LOC105277290
Obir_9S_LOC105277290
Obir_9S_LOC105277285
Obir_9S_LOC105277286
Obir_9S_LOC109610840
Obir_9S_LOC105277289
Mpha_9_XP_036144419.1
Mpha_9_XP_012529710.1
Mpha_9_XP_036144417.1
Mpha_9_XP_036144418.1
Cflo_9_XP_011263149.2
Cflo_9_XP_011263150.2
Cflo_9_XP_011263148.2
Mpha_9_XP_036144986.1_2
Mpha_9_XP_012536463.1
Mpha_9_XP_028049478.1
Mpha_9_XP_012541379.2
Mpha_9_XP_012521731.2
Cflo_9_XP_011263155.2
Cflo_9_XP_019884430.2
Cflo_9_XP_011263154.1
Cflo_9_XP_025263872.1
Hsal_9_XP_011140514.1
Hsal_9_XP_011149378.2
Hsal_9_XP_025156106.1
Hsal_9_XP_011140511.2
Hsal_9_XP_011140512.1
Cflo_9_XP_025263913.1
Cflo_9_XP_025263781.1
Cflo_9_XP_025263910.1
Cflo_9_XP_025263911.1
Cflo_9_XP_025263955.1
Cflo_9_XP_025263909.1
Cflo_9_XP_025263961.1
Cflo_9_XP_011266968.2
Cflo_9_XP_025263908.1
Cflo_9_XP_025263779.1
Cflo_9_XP_025263907.1
Mpha_9_XP_012529713.1
Mpha_9_XP_012540006.1
Mpha_9_XP_012535425.1
Mpha_9_XP_012529711.2
Mpha_9_XP_036144420.1
Mpha_9_XP_036144421.1
Mpha_9_XP_036144986.1_0
Mpha_9_XP_036142689.1
Obir_9S_LOC105277277
Obir_9S_LOC105277277
Obir_9S_LOC105277278
Obir_9S_LOC105277278
Obir_9S_LOC105277280
Obir_9S_LOC105277280
Obir_9S_LOC105277288
Obir_9S_LOC105277281
Cflo_9_XP_025263901.1
Cflo_9_XP_025263906.1
Cflo_9_XP_025263902.1
Cflo_9_XP_025263903.1
Cflo_9_XP_025263904.1
Cflo_9_XP_011266965.2
Mpha_9_XP_012541825.2
Mpha_9_XP_012533028.1
Hsal_9_XP_011146998.1
Amel_9Q1
Amel_9Q3
Amel_9Q2
Amel_9R1
Amel_9S1
Cflo_9_XP_011268180.2
Mpha_9_XP_012526852.1
Mpha_9_XP_012526854.1
Mpha_9_XP_012526853.1
Mpha_9_XP_012537471.1
Mpha_9_XP_036150068.1
Obir_9Chr1
Obir_9Chr5
Obir_9Chr9
Hsal_9_XP_011152942.1
Hsal_9_XP_025162057.1
Hsal_9_XP_025162058.1
Hsal_9_XP_019698679.2
Cflo_9_XP_011268181.2
Nvit_9AG1
Nvit_9AG2
Nvit_9AG6
Nvit_9AG7
Nvit_9AG3
Nvit_9AG4
Nvit_9AG5
Nvit_9AH1
Nvit_9AH2
Nvit_9AH3
Nvit_9AH4
Nvit_9AH5
Nvit_9AH6
Nvit_9AH7
0.3

Figure S7. **Maximum likelihood phylogeny of CYP9 protein sequences derived from hymenopteran genomes, related to Figure 7.** Amel: *Apis mellifera*, Nvit: *Nasonia vitripennis*, Obir: *Ooceraea biroi*, Hsal: *Harpegnathos saltator*, Cflo: *Camponotus floridanus*, Mpha: *Monomorium pharaonis*. Each ortholog group is highlighted, and the numbers to the right give the number of copies in each species. All Chromosome 13 *CYP9*s in *O. biroi* fall within the loss of heterozygosity region and span five ortholog groups (CYP9P, CYP9S 'A'-'D'). All duplicate members of each CYP9 family are monophyletic in *O. biroi* as well as other ant species, and the *O. biroi* genes have a phylogeny concordant with their spatial organization (Figure 7B), indicating that a combination of gene conversion, duplication and deletion, and/or purifying selection has prevented the members of each ortholog group from diverging in sequence, resulting in a pattern of coordinated evolution. Node labels represent bootstrap support from 100 replicates, and the scale bar represents 30% divergence at informative sites. Nodes with less than 80% bootstrap support were collapsed.

Clonal Line	Colonies	Individuals	Microsatellite locus					
			DK371	ES177	D8Z0W	D8M16	D4XW2	ER4IH
Line A	19	27	192/192	219/219	231/231	180/188	214/220	227/227
Line A	4	5	192/192	219/219	231/231	180/188	220/220	227/227
Line B	31	31	192/192	216/219	231/233	180/182	217/220	227/230
Line B	9	9	192/192	219/219	231/233	180/182	217/220	227/230
Line B	3	3	192/192	216/216	231/233	180/182	217/220	227/230
Line B	2	2	192/192	216/219	231/231	180/182	220/220	227/230
Line B	1	1	192/192	216/216	231/233	180/182	217/220	227/227
Line B	1	1	186/192	219/221	229/231	176/180	214/220	227/230
Line B	1	1	186/192	219/221	229/231	176/180	214/220	227/227
Line C	8	8	186/192	213/213	229/231	176/199	214/214	236/236
Line D	1	1	186/186	213/213	231/231	176/197	214/220	227/236
Line D	1	1	192/192	219/219	231/231	176/197	214/220	227/236
Queen-like mutant	1	6	192/192	219/219	231/231	180/188	214/220	227/227
Queen-like mutant	1	1	192/192	219/219	231/231	180/188	214/214	227/227

Table S1. Microsatellite genotypes for queen-like mutants and the four invasive clonal lines of *O. biroi*, related to Figure 1. Queen-like mutants belong to Clonal Line A and, like wild-types, are parthenogenetic.

Supplementary References

S1. Connallon, T., and Hodgins, K.A. (2021). Allen Orr and the genetics of adaptation. Evolution *75*, 2624–2640.

S2. Kuhn, A., Darras, H., and Aron, S. (2018). Phenotypic plasticity in an ant with strong caste – genotype association. Biol. Lett. *14*, 20170705.

S3. Oxley, P.R., Ji, L., Fetter-Pruneda, I., McKenzie, S.K., Li, C., Hu, H., Zhang, G., and Kronauer, D.J.C. (2014). The genome of the clonal raider ant *Cerapachys biroi*. Curr. Biol. *24*, 451–458.

S4. Dobata, S., Sasaki, T., Mori, H., Hasegawa, E., Shimada, M., and Tsuji, K. (2009). Cheater genotypes in the parthenogenetic ant *Pristomyrmex punctatus*. Proc. Biol. Sci. *276*, 567–574.

S5. Fournier, D., Estoup, A., Orivel, J., Foucaud, J., Jourdan, H., Le Breton, J., and Keller, L. (2005). Clonal reproduction by males and females in the little fire ant. Nature *435*, 1230–1234.

S6. Trible, W., and Kronauer, D.J.C. (2020). Hourglass model for developmental evolution of ant castes. Trends Ecol. Evol. *36*, 100–103.

S7. Trible, W., and Kronauer, D.J.C. (2017). Caste development and evolution in ants: it's all about size. J. Exp. Biol. *220*, 53–62.

S8. Ravary, F., and Jaisson, P. (2004). Absence of individual sterility in thelytokous colonies of the ant *Cerapachys biroi* Forel (Formicidae, Cerapachyinae). Insectes Soc. *51*, 67–73.

S9. Rabeling, C. (2021). Social Parasitism. Encycl. Soc. Insects, 836–858.

S10. Rabeling, C., Schultz, T.R., Pierce, N.E., and Bacci, M. (2014). A social parasite evolved reproductive isolation from its fungus-growing ant host in sympatry. Curr. Biol. *24*, 2047–2052.

S11. Schwander, T., Lo, N., Beekman, M., Oldroyd, B.P., and Keller, L. (2010). Nature versus nurture in social insect caste differentiation. Trends Ecol. Evol. *25*, 275–82.

S12. Tsuji, K., and Dobata, S. (2011). Social cancer and the biology of the clonal ant *Pristomyrmex punctatus* (Hymenoptera: Formicidae). Myrmecological News *15*, 91–99.

S13. Wang, M. (2003). A monographic revision of the ant genus *Pristomyrmex* (Hymenoptera: Formicidae). Bull. Museum Comp. Zool. *157*, 383–542.

S14. Trible, W., and Kronauer, D.J.C. (2021). Ant caste evo-devo: Size predicts caste (almost) perfectly. Trends Ecol. Evol. *36*, 671–673.

S15. McInnes, D.A., and Tschinkel, W.R. (1995). Queen dimorphism and reproductive strategies in the fire ant *Solenopsis geminata* (Hymenoptera: Formicidae). Behav. Ecol. Sociobiol. *36*, 367–375.

S16. Rüppell, O., Heinze, J., and Hölldobler, B. (1998). Size-dimorphism in the queens of the North American ant *Leptothorax rugatulus* (Emery). Insectes Soc. *45*, 67–77.

S17. Borowiec, M.L., Cover, S.P., and Rabeling, C. (2021). The evolution of social parasitism in *Formica* ants revealed by a global phylogeny. Proc. Natl. Acad. Sci. *118*, e2026029118.

S18. Wilson, E.O. (1984). Tropical social parasites in the ant genus *Pheidole*, with an analysis of the anatomical parasitic syndrome (Hymenoptera: Formicidae). Insectes Soc. *31*, 316–334.

S19. Cole, A.C. (1965). Discovery of the worker caste of *Pheidole* (P.) *inquilina*, new combination (Hymenoptera: Formicidae). Ann. Entomol. Soc. Am. *58*, 173–175.

S20. Sumner, S., Nash, D.R., and Boomsma, J.J. (2003). The adaptive significance of inquiline

parasite workers. Proc. Biol. Sci. *270*, 1315–1322.

S21. Yang, S., Wang, L., Huang, J., Zhang, X., Yuan, Y., Chen, J.Q., Hurst, L.D., and Tian, D. (2015). Parent-progeny sequencing indicates higher mutation rates in heterozygotes. Nature *523*, 463–467.

S22. Liu, H., Jia, Y., Sun, X., Tian, D., Hurst, L.D., and Yang, S. (2017). Direct determination of the mutation rate in the bumblebee reveals evidence for weak recombination-associated mutation and an approximate rate constancy in insects. Mol. Biol. Evol. *34*, 119–130.

S23. Dermauw, W., Van Leeuwen, T., and Feyereisen, R. (2020). Diversity and evolution of the P450 family in arthropods. Insect Biochem. Mol. Biol. *127*, 103490.

S24. Peeters, C., and Crozier, R.H. (1988). Caste and reproduction in ants: not all mated egg-layers are "queens." Psyche *95*, 283–288.

S25. Yagound, B., Dogantzis, K.A., Zayed, A., Lim, J., Broekhuyse, P., Remnant, E.J., Beekman, M., Allsopp, M.H., Aamidor, S.E., Dim, O., *et al.* (2020). A single gene causes thelytokous parthenogenesis, the defining feature of the Cape honeybee *Apis mellifera capensis*. Curr. Biol. *30*, 1–12.

S26. Fresneau, D., and Dupuy, P. (1988). A study of polyethism in a ponerine ant: *Neoponera apicalis* (Hymenoptera, Formicidae). Anim. Behav. *36*, 1389–1399.

S27. Glastad, K.M., Graham, R.J., Ju, L., Roessler, J., Brady, C.M., and Berger, S.L. (2020). Epigenetic regulator CoREST controls social behavior in ants. Mol. Cell *77*, 338-351.

S28. Rey, O., Facon, B., Foucaud, J., Loiseau, A., and Estoup, A. (2013). Androgenesis is a maternal trait in the invasive ant *Wasmannia auropunctata*. Proc. R. Soc. B Biol. Sci. *280*, 20131181.

S29. Lacy, K.D., Shoemaker, D., and Ross, K.G. (2019). Joint evolution of asexuality and queen number in an ant. Curr. Biol. *29*, 1394–1400.
